# Circularity and self-cleavage as a strategy for the emergence of a chromosome in the RNA-based protocell

**DOI:** 10.1186/1745-6150-8-21

**Published:** 2013-08-23

**Authors:** Wentao Ma, Chunwu Yu, Wentao Zhang

**Affiliations:** 1 College of Life Sciences, Wuhan University, Wuhan 430072, P.R. China; 2 College of Computer Sciences, Wuhan University, Wuhan 430072, P.R. China

## Abstract

**Background:**

It is now popularly accepted that an “RNA world” existed in early evolution. During division of RNA-based protocells, random distribution of individual genes (simultaneously as ribozymes) between offspring might have resulted in gene loss, especially when the number of gene types increased. Therefore, the emergence of a chromosome carrying linked genes was critical for the prosperity of the RNA world. However, there were quite a few immediate difficulties for this event to occur. For example, a chromosome would be much longer than individual genes, and thus more likely to degrade and less likely to replicate completely; the copying of the chromosome might start at middle sites and be only partial; and, without a complex transcription mechanism, the synthesis of distinct ribozymes would become problematic.

**Results:**

Inspired by features of viroids, which have been suggested as “living fossils” of the RNA world, we supposed that these difficulties could have been overcome if the chromosome adopted a circular form and small, self-cleaving ribozymes (e.g. the hammer head ribozymes) resided at the sites between genes. Computer simulation using a Monte-Carlo method was conducted to investigate this hypothesis. The simulation shows that an RNA chromosome can spread (increase in quantity and be sustained) in the system if it is a circular one and its linear “transcripts” are readily broken at the sites between genes; the chromosome works as genetic material and ribozymes “coded” by it serve as functional molecules; and both circularity and self-cleavage are important for the spread of the chromosome.

**Conclusions:**

In the RNA world, circularity and self-cleavage may have been adopted as a strategy to overcome the immediate difficulties for the emergence of a chromosome (with linked genes). The strategy suggested here is very simple and likely to have been used in this early stage of evolution. By demonstrating the possibility of the emergence of an RNA chromosome, this study opens on the prospect of a prosperous RNA world, populated by RNA-based protocells with a number of genes, showing complicated functions.

**Reviewers:**

This article was reviewed by Sergei Kazakov (nominated by Laura Landweber), Nobuto Takeuchi (nominated by Anthony Poole), and Eugene Koonin.

## Background

In modern cells, genes coding for different proteins can exist in the same DNA molecule (chromosome) and are replicated together. This “arrangement” is important to avoid “gene loss” during cell division. In prokaryotes there is only one chromosome. Although there are more chromosomes in eukaryotes, their numbers are still quite limited compared with the number of genes. Indeed, even for appropriate distribution of this limited number of chromosomes between offspring cells, a very complicated mechanism has evolved. It is now popularly accepted that in the early evolution of life, there was a stage called “the RNA world” [1,2], in which RNA was the carrier of both genes and functions. Then, could different genes exist in one chromosome at this stage? This is a question that concerns the possibility of the success of a prosperous RNA world. When more genes evolved, the gene loss would become a more serious problem (an offspring protocell would be more likely to lack some genes).

Theoretical studies have indicated that replicators with different functions may coexist/cooperate in a cell-like container [3-5]. A replicator can be deemed to be an abstract representation of an RNA molecule. A container with functional replicators has been described as “a bag of genes” [6]. Recently we reported a computer simulation study using a more concrete model that indicated that ribozymes (simultaneously as genes) with different functions may coexist/cooperate in RNA-based protocells [7]. Further, it has been realized that the emergence of a chromosome with linked genes may have a selective advantage to prevent the risk of gene loss during the division of the protocells [6,8]. Another advantage may be associated with the labor division of template and catalysts: a chromosome would work specially as template (in the replication) and ribozymes would work as catalysts. Efficient ribozymes need to have strong, complicated structures, but this would impede their role as templates. Linked “ribozyme domains” within the chromosome might interfere with each other, making them difficult to adopt the “right” structures as they could in their free forms. Therefore, in the long run, the labor division may favor the emergence of more efficient ribozymes. This idea, concerning the advantage of labor division between template and catalysts, is somewhat similar to the one proposed earlier [9], in which the emergence of DNA in the RNA world was discussed.

However, there would be quite a few difficulties for the emergence of a chromosome. First, the RNA chromosome would be much longer than the single RNA genes and would thus run much more risk of degradation (chain breaking). Second, because of the significantly longer chain, its full replication would be much slower. Certainly, as mentioned above, the chromosome can be expected to be a better template (thus favoring the replication) because of the interference of individual “ribozyme domains” with each other in folding. However, the effect of this factor may be still limited (perhaps sequence-dependent). Third, the copying of the chromosome may start at some sites in the middle of the chain, and thereby would not result in full replication. Fourth, at this early stage of evolution, without a complicated mechanism of “transcription” (for example, promoters and ending signals), the synthesis of distinct gene products from the chromosome would become problematic. Compared with these difficulties, the selective advantages mentioned above are apparently more indirect. Considering that evolution is continuous and cannot “see advantages in the future”, these difficulties would have to have been be overcome first.

A simple strategy is implied in certain small plant pathogenic RNAs (viroids or viroid-like satellite RNAs), which were suggested to be plausible candidates as living fossils of the RNA world [10]. A viroid has a single-stranded, circular RNA chromosome as its “genome” (only about 250–400 nt long and without any genes coding for proteins) that replicates by a rolling-circle mechanism [11,12]. In its replication, a long linear molecule that contains multiple copies of the genome is produced, which is then cleaved to generate individual copies by a host RNase or a small catalytic segment (the hammerhead ribozyme) within the viroid genome. The latter way, namely self-cleavage by catalytic RNA, should be the more ancient one. These features of circularity and self-cleavage are also shared by viroid-like satellite RNAs [13,14].

The first three difficulties mentioned above can be overcome (or at least alleviated) by circularity. First, by circularity, the chromosome could evade the degradation starting from its chain ends, and this would compensate to some degree the disadvantage concerning degradation due to its longer chain. The end-degradation may be caused by the easier breaking of terminal phosphodiester bonds than internal ones, or by the spontaneous decay of terminal nucleotide residues (see the Discussion section for detailed explanations). If such end-degradation was strong, such compensation may be expected to be significant. Second, the circular topology is expected to further impede (sterically hinder) the folding of distinct “ribozyme domains” in the chromosome, making the chromosome more suitable to act as a template in the replication. Third, there would be no issue concerning the starting point of copying [10]. The fourth difficulty can be overcome by self-cleavage. If the “sense strand” of the chromosome tends to be broken at the sites between genes, corresponding ribozymes may be produced this way. A hammerhead ribozyme catalyzing self-cleavage may have existed at these sites. The hammerhead ribozyme, the smallest ribozyme found in living beings [15], has a highly conserved sequence and its DNA motif exists ubiquitously in the genomes of modern organisms [16,17]. In particular, the motif has been found between genes in bacterial genomes [16]. Probably, this emerged initially in the RNA world, where it participated in the production of distinct ribozymes from the RNA chromosome.

**Figure 1:**
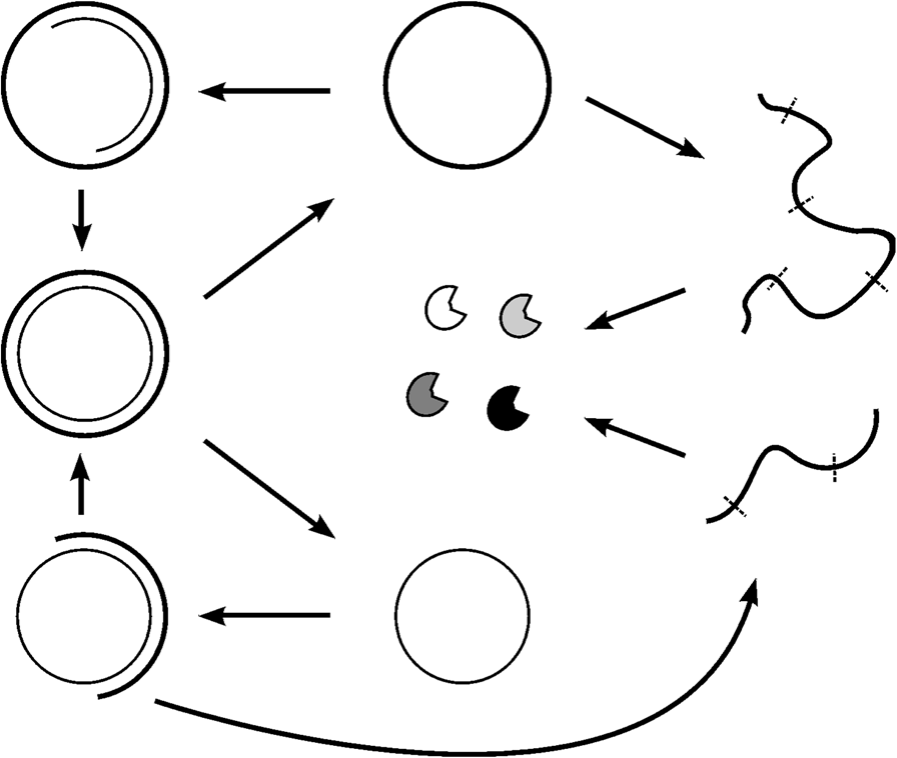
**Circularity and self-cleavage as a strategy for the primary chromosome in the RNA world.** Thick lines represent the sense chain, and thin lines represent the antisense chain. The linear RNAs, which arise from the spontaneous break of the sense chain or partial copying of the antisense chain, may be readily broken at sites (labeled by virtual bands) between the linked genes. This feature may be implemented by the participation of short self-cleaving ribozymes residing at these sites. The products of the cleavage are the RNAs that may fold into various ribozymes (crescent-shapes). Note: the self-cleaving effect would not occur in the circular chromosome because steric constraints would inhibit the folding and function of the embedded self-cleaving ribozymes, consistent with the situations in viroids [11,12].

In order to determine the plausibility of this deduction, we conducted a computer simulation study. The simulation shows that an RNA chromosome can spread (increase in quantity and be sustained) in the system when it adopts a circular form and its linear “transcripts” (corresponding to the “sense strand”) is readily broken at the sites between genes. Here we suggest that circularity plus self-cleavage could have been used as a strategy for the emergence of a chromosome in the RNA-based protocell (Figure 1).

## Results

We investigated a hypothetical RNA chromosome that included four genes coding for a replicase ribozyme (“Rep”; catalyzing the template-directed replication of RNA), a nucleotide synthetase ribozyme (“Nsr”), a nucleotide precursor synthetase ribozyme, (“Npsr”) and an amphiphile (membrane component) synthetase ribozyme (“Asr”). The approach of the simulation is similar to those used in our previous work studying Rep [18], Nsr [19], Asr [20], and their cooperation [7]. The introduction of a new functional ribozyme, Npsr, was aimed at increasing the number of genes in the chromosome, as a more representative form of a chromosome with linked genes.

The simulation was based on a Monte-Carlo model, in which each event in the system may occur with some probability in a specific time step (Table 1). The circularity of the chromosome per se and three parameters used in the model (*P*_*CRTT*_, *P*_*LRTT*_, and *F*_*IB*_ described below) are associated with the topic of this study. The chromosome (as a circular RNA) may become a template with *P*_*CRTT*_ (the probability of a circular RNA turning to a template), which should be higher than *P*_*LRTT*_ (the probability of a linear RNA turning to a template), with which individual ribozymes (as linear RNAs) may become templates. Linear “transcripts” (i.e., linear RNAs that result from partial copying of the antisense chain or the spontaneous break of the sense chain of the circular chromosome, Figure 1) would be easier to break at the sites between genes than at other sites, by *F*_*IB*_ (factor for intermediate RNA breaking at sites between genes) times. This assumption represents the self-cleaving effect. It should be noted that the self-cleaving effect would not occur in the circular chromosome because steric constraints would inhibit the folding and function of the embedded self-cleaving ribozymes, as well as that of the ribozymes corresponding to those “genes”. This is consistent with the findings in viroids, wherein the embedded hammerhead ribozyme is, in fact, inactive in the circular RNA chromosome, but would work during the rolling-circle replication, when it resides in the long linear RNA, between the genome copies [11,12]. The model is described in more details in the Methods section.

**Table 1:** Parameters used in the Monte Carlo simulations

**Probabilities**	**Descriptions**	**Values** ^*^
*P*_*AD*_	Amphiphile decaying into its precursor (out of membrane)	5 × 10^-4^
*P*_*ADM*_	Amphiphile decaying into its precursor within membrane	5 × 10^-5^
*P*_*AF*_	Amphiphile forming from its precursor (not catalyzed)	5 × 10^-4^
*P*_*AFR*_	Amphiphile forming from its precursor (catalyzed by Asr)	0.9
*P*_*AJM*_	Amphiphile joining membrane	0.9
*P*_*ALM*_	Amphiphile leaving membrane	5 × 10^-5^
*P*_*APP*_	Amphiphile precursor permeating membrane	0.05
*P*_*AT*_	RNA attracting nucleotides/oligomers by base-pairing	0.2
*P*_*BB*_	Phosphodiester bond breaking in an RNA chain	2 × 10^-6^
*P*_*CB*_	Protocell breaking	1 × 10^-5^
*P*_*CD*_	Protocell dividing	0.02
*P*_*CF*_	Protocell fusing	5 × 10^-4^
*P*_*CRTT*_	A circular RNA turning to a template	0.9
*P*_*EL*_	End-to-end ligation of an RNA chain (cyclization)	1 × 10^-7^
*P*_*FLR*_	Ligating with false base-pairing on template (by Rep)	0.01
*P*_*FP*_	False base-pairing in RNA attracting nucleotides/oligomers	0.01
*P*_*LRTT*_	A linear RNA turning to a template (i.e., unfolding)	0.01
*P*_*MC*_	Movement of a protocell	0.05
*P*_*MF*_	Membrane forming	0.1
*P*_*MV*_	Movement of a nucleotide, amphiphile or their precursors	0.5
*P*_*ND*_	Nucleotide decaying into its precursor	5 × 10^-4^
*P*_*NDE*_	Nucleotide decaying into its precursor at RNA’s chain end	2 × 10^-5^
*P*_*NF*_	Nucleotide forming from its precursor (not catalyzed)	5 × 10^-4^
*P*_*NFR*_	Nucleotide forming from its precursor (catalyzed by Nsr)	0.9
*P*_*NPD*_	Nucleotide precursor decaying into its precursor	5 × 10^-4^
*P*_*NPF*_	Nucleotide precursor forming from its precursor (not catalyzed)	5 × 10^-4^
*P*_*NPFR*_	Nucleotide precursor forming from its precursor (catalyzed by Npsr)	0.9
*P*_*NPP*_	Nucleotide precursor permeating membrane	0.01
*P*_*NPPP*_	Nucleotide precursor’s precursor permeating membrane	0.2
*P*_*RB*_	Rep binding onto an RNA template	0.9
*P*_*RD*_	Rep dropping from an RNA template	0.9
*P*_*RL*_	Random ligation of nucleotides and RNA	1 × 10^-7^
*P*_*SP*_	Separation of a base pair	0.5
*P*_*TL*_	Template-directed ligation (not catalyzed)	5 × 10^-4^
*P*_*TLR*_	Template-directed ligation (catalyzed by Rep)	0.9
**Others**	**Descriptions**	Values ^*^
*F*_*DE*_	Factor for the effect of Donnan’s equilibrium [28]	5
*F*_*DO*_	Factor for the degradation/decay of molecules out of protocells	20
*F*_*IB*_	Factor for intermediate RNA breaking (at sites between genes)	100
*F*_*OP*_	Factor for the effect of osmotic pressure [27]	5
*L*_*AM*_	Lower limit of amphiphiles to form protocell membrane	600
*N*	The system surface is defined as an N × N grid.	40
*T*_*APB*_	Total amphiphile precursors introduced in the beginning	6 × 10^4^
*T*_*NPPB*_	Total nucleotide precursors’ precursors introduced in the beginning	8 × 10^4^

**Figure 2:**
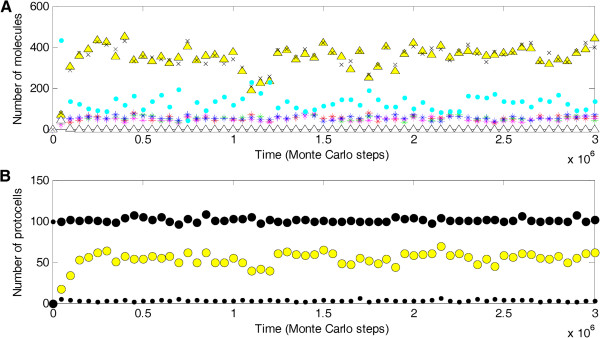
**Representative case showing the spread of the chromosome. (A)** At the molecular level. The characteristic domains for the ribozymes (stars) Rep (red), Nsr (green), Npsr (magenta) and Asr (blue) are assumed to be “GAGUCUCU”, “GCUCGUAU”, “GGUUCGAU” and “GCGACUUU”, respectively. The sense chain of the chromosome (yellow triangles) has a sequence of these four domains in a tandem and circular way. The antisense chain of the chromosome (x-shapes) is complementary to the sense chain. Linear RNA chains are assumed to break more readily (involving the factor *F*_*IB*_) at sites after U and before G than other sites, which represents the self-cleavage effect between genes (“U-G” sites are avoided within the assumed gene domains mentioned above). The control (white triangles) has a circular chain with length identical to the chromosome, but without any gene domains (“GCCUUAGUGGACUCUUGAUAGCGUGGAAGUCU”). The number of nucleotide precursors’ precursors (cyan dots) is represented in a 1/64 scale (i.e., quotients in measurement of the mass of a sense chain and an antisense chain of the chromosome, which have 32 nucleotide residues each). **(B)** At the cellular level. Black circles represent total protocells. Yellow circles represent protocells containing at least one molecule of the chromosome (sense chain). Amphiphile precursors (black dots) are represented in a 1/600 scale (i.e., quotients in measurement of the lower limit of amphiphiles to form a protocell membrane, *L*_*AM*_). The parameter values for this case are listed in Table 1. The random seed is 9.

* This set of parameter values is for the case shown in Figure 2, and also represents the common parameter list that was used for the parameter analysis in Figure 4 and in the Additional file 1: Figures S2-S5.

Nucleotide precursors’ precursors and amphiphile precursors were introduced at the initial step of the simulation as raw materials. Some protocells containing a few molecules of the ribozymes (Rep, Nsr, Npsr and Asr) and the sense chain of the circular chromosome were inoculated at an early step. By testing parameter values with the considerations associated with the three parameters mentioned above (most of the other parameters have been used in our previous studies [7,18-20] and were, in general, set accordingly in the present study; see also the Methods section for some considerations on the logical relations of the parameters), it was found that the chromosome might spread in the system. Figure 2 shows a typical case of the spread and the parameter set that was used in this case (Table 1) represents a typical set of parameter values that support the spread of the chromosome. However, it should be noted that, as in our previous studies that also used models of this type defined by numerical probabilities, this does not mean that the result is sensitive to detailed values in the typical set. In fact, the spread of the chromosome is quite robust against moderate variations of these values. Certainly, the parameter variations may favor or disfavor the spread (as discussed below).

The number of chromosome molecules (yellow triangles as the sense chain and “x-shapes” as the antisense chain, in Figure 2A) increases and finally reaches equilibrium. The increase and decrease in the number of chromosome molecules at the equilibrium is generally opposite to that of the raw materials (nucleotide precursors’ precursors, cyan dots). When the antisense chain was inoculated instead of the sense chain, the results were similar (in the Additional file 1: Figure S1). While the chromosome molecules spread to a high balance level, the ribozymes (stars) remained at a lower balance level. The likely reason for this is that, because there is not a mechanism like transcription, the ribozymes are only byproducts that result from the replication of the chromosome molecule (via self-cleaving, see Figure 1). Accompanying the spread of the chromosome, protocells containing chromosome molecules (yellow circles in Figure 2B) became the major portion of total protocells (black circles).

**Figure 3:**
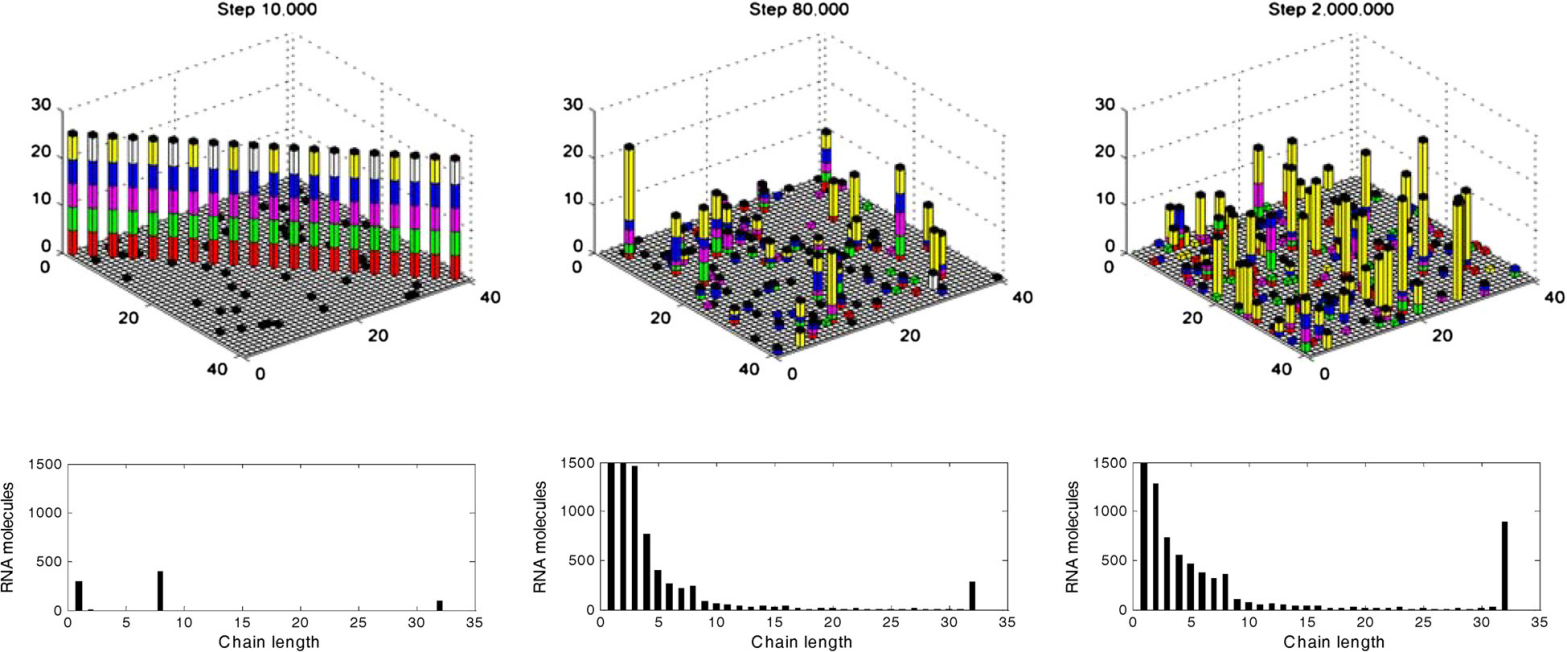
**The spatial distribution and the chain length distribution during the spread of the chromosome (for the case shown in Figure**2**).** (***Top row***) The spatial distribution. The horizontal plane is the *N* × *N* grid. A bar in a grid room represents the number of ribozymes, Rep (red), Nsr (green), Npsr (magenta) and Asr (blue), and the chromosome (yellow) / the control (white), in a stacked form. A black cap on a bar represents that the grid room is occupied by a protocell and thus the RNA molecules are in the protocell (a sole cap means an “empty” protocell). At step 1 × 10^4^ (the left panel), 10 grid rooms at the diagonal of the grid were each inoculated with an protocell containing 5 molecules of the ribozymes and the sense chain of the chromosome; 10 other rooms, also at the diagonal of the grid, were each inoculated with a protocell containing 5 molecules of the ribozymes and the control. The empty protocells at this step result from the spread of the 10 empty protocells inoculated at step 1 × 10^3^ (not shown here). The middle panel shows the spatial distribution at step 8 × 10^4^, and the right panel at step 2 × 10^6^. (***Bottom row***) The chain-length distribution of RNA molecules. The steps for the left, middle and right panels correspond to those shown in the top row. In the middle panel, the numbers of monomers (25,775) and dimers (3,389) are not fully represented; in the right panel, the number of monomers (9,287) is not fully represented.

For the representative case in Figure 2, the spatial distributions of the chromosome and ribozymes at the inoculation step (1 × 10^4^), at a step during the spread (8 × 10^4^), and at a step after the spread (2 × 10^6^) are shown in the top row of Figure 3; the chain length distribution of RNA molecules in the system at the corresponding steps are shown in the bottom row of Figure 3. In the bottom-left panel (step 1 × 10^4^), the left bar represents the monomers (nucleotides) that were formed from nucleotide precursors, which in turn formed from nucleotide precursors’ precursors introduced at the initial step, both by non-enzymatic synthesis; the middle bar represents the ribozymes (8 nt long, see the legend to Figure 2) that were inoculated at this step; and the right bar represent the circular chromosome (32 nt long). In the bottom-middle panel (step 8 × 10^4^), the 32-nt RNAs increase, probably representing the chromosome, along with a few variants caused by inaccurate replication (associated with *P*_*FP*_ and *P*_*FLR*_ in the model). For the RNAs shorter than 32 nt, in general, the molecule number increased as the chain length decreased, reflecting the effect of RNA degradation. The 8-nt RNAs are more than 7-nt RNAs (opposite to the general tendency), reflecting the effect of assumption that the sites between genes are easier to break than other sites (associated with *F*_*IB*_). In the bottom-right panel (step 2 × 10^6^), the situation is similar, except that the 32-nt RNAs increase further.

**Figure 4:**
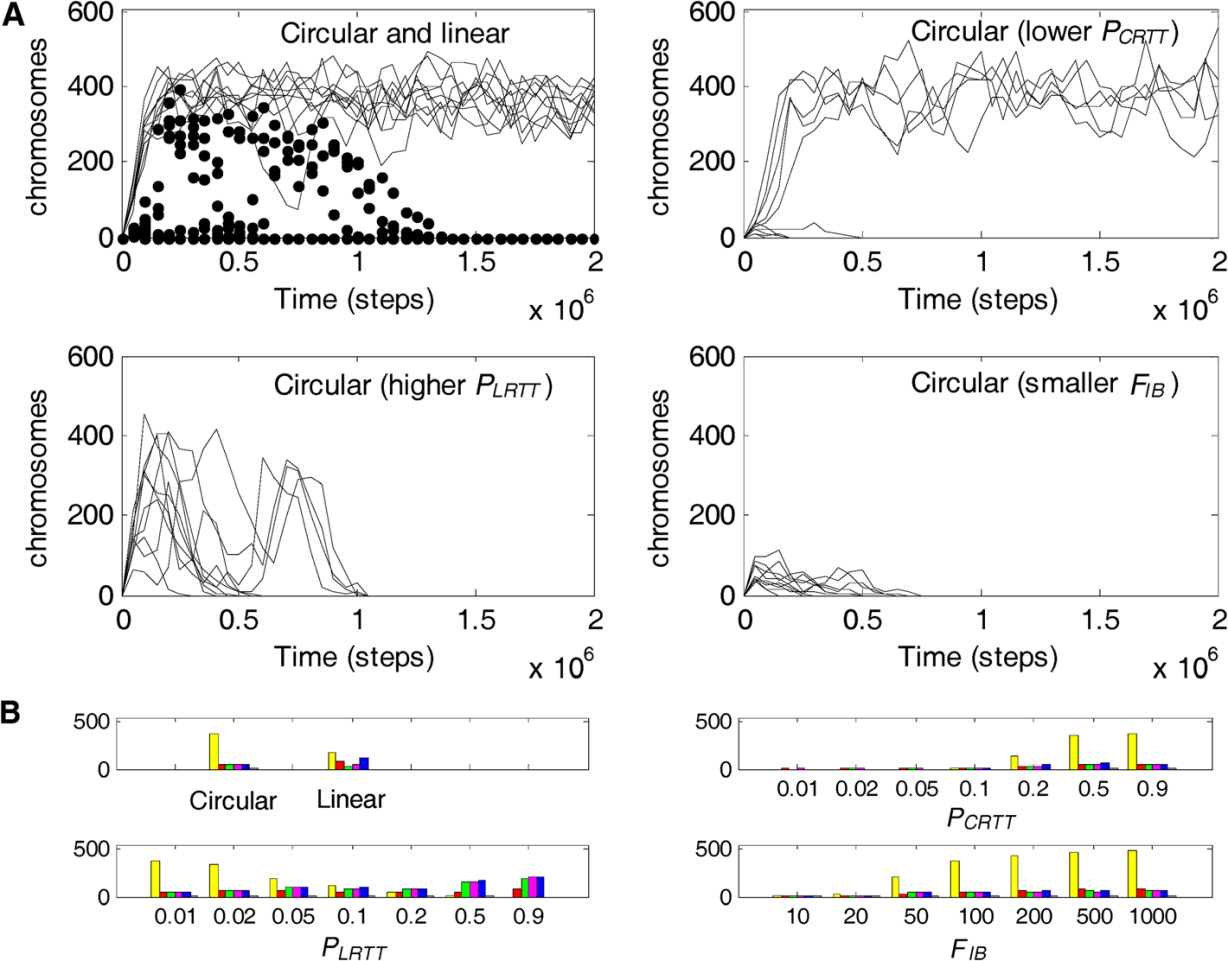
**Analysis of the roles of circularity and self-cleavage on the spread of the chromosome. (A)** The vertical axis represents the molecule number of the chromosome (the sense chain). Random seeds 1–10 were used to initiate the 10 different cases. In the cases shown in the top-left panel, all parameter values are set according to the common parameter list (identical to those used for the case shown in Figure 2, and listed in Table 1; wherein, *P*_*CRTT*_ = 0.9, *P*_*LRTT*_ = 0.01 and *F*_*IB*_ = 100). Lines represent the cases for the circular chromosome, and dots for the linear chromosome. The top-right, bottom-left and bottom right panels, shows the cases for the circular chromosome assuming that *P*_*CRTT*_ decreased to 0.2, *P*_*LRTT*_ increased to 0.05, and *F*_*IB*_ decreased to 20, respectively. **(B)** A tick on a horizontal axis denotes a value of the corresponding parameter (except for the top-left panel). Random seeds 1−100 were used to initiate 100 different cases adopting such a parameter value, whereas values of the other parameters are set according to the common parameter list (Table 1). For the cases in the top-left panel, all parameters are set according to this list, but the cases for the circular chromosome and the linear chromosome are compared. The bars in a bar group represent the molecule numbers (averaged over the 100 cases) of the chromosome (the sense chain; yellow), Rep (red), Nsr (green), Nspr (magenta), Asr (blue) and the control (grey) recorded at step 2 × 10^5^. This step was adopted from experience to show the influence of the parameters clearly and also with consideration for the computational (time) cost.

Further analysis was done to explore the influence of the circularity and self-cleavage on the spread of the chromosome. 10 different random seeds were used to initiate 10 simulation cases for each situation described below. For the 10 cases (lines) in the top-left panel of Figure 4A, we adopt parameter values that were identical to those for the case shown in Figure 2 (see Table 1, where, *P*_*CRTT*_ = 0.9, *P*_*LRTT*_ = 0.01 and *F*_*IB*_ = 100). In all 10 cases, the chromosome (represented by the sense chain) spread. When the chromosome was assumed to be linear, it did not spread to a balance level (dots). In these cases, the probability of the chromosome becoming a template was also assumed to be high (the same as the assumption for the circular chromosome, i.e., equaling to 0.9). The only difference was that, in these cases, RNA end-degradation (associated with *P*_*NDE*_) would act on the linear chromosome molecules. This result demonstrates the importance of circularity for the chromosome to avoid end-degradation (the advantage of circularity to alleviate the first difficulty, as described in the Introduction). For the circular chromosome, when *P*_*CRTT*_ was changed from 0.9 to 0.2, the spread occurred only in 5 of the 10 cases (Figure 4A, top-right panel). This shows that a high probability for the chromosome becoming a template is important for its spread. More importantly, when *P*_*LRTT*_ was changed from 0.01 to 0.05, whereas *P*_*CRTT*_ remained at 0.9, none of the ten cases could spread to the equilibrium (Figure 4A, bottom-left panel). This shows that a significantly higher probability of the circular chromosome becoming a template compared with that of linear ribozymes would be important for the spread of the chromosome (the advantage of circularity to alleviate the second difficulty). The advantage of circularity to overcome the third difficulty for the emergence of the chromosome, i.e., evading the partial replication starting at middle sites of a linear chromosome [10], is difficult to judge from the present model. On the other hand, there may be some reasonable arguments against such an advantage. For example, it may be argued that the process of replication in the RNA world may not have been unidirectional, so starting anywhere along a linear chain might have provided equal probability that the chain would be replicated (in full). Therefore, leaving this possible advantage not judged is not necessarily a deficiency. When *F*_*IB*_ was changed from 100 to 20 (i.e., the rate of RNA breaking at the sites between genes is 20 times higher than other sites), the spread of the chromosome was already seriously impeded (Figure 4A, bottom-right panel). This shows that a significantly higher tendency of the inter-gene chain breaking is important for the spread of the chromosome, which supports our hypothesis that self-cleavage by the hammerhead ribozymes residing between genes may play an important role in overcoming the fourth difficulty, namely., the synthesis of distinct gene products from the chromosome.

To confirm our judgments on the role of circularity and self-cleavage, a more extensive analysis was conducted. The influence of the parameters on the spread of the chromosome was determined at a broader range of values. For each value, 100 different random seeds were used to initiate 100 simulation cases and the numbers of chromosome molecules (represented by the sense chain) and ribozymes at step 2 × 10^5^ were recorded and averaged. The average numbers were drawn as bars (yellow: the chromosome; red: Rep; green: Nsr; magenta: Nspr; blue: Asr; grey: the control) in Figure 4B. The results support the previous judgments. First, circularity to avoid end-degradation favors the spread of the chromosome (Figure 4B, top-left panel). Next, a high probability of the chromosome becoming a template is important (Figure 4B, top-right panel). In addition, if the probability of the chromosome becoming a template is high (*P*_*CRTT*_ = 0.9), but the probability of the ribozymes becoming a template is not low enough (Figure 4B, bottom-left panel, *P*_*LRTT*_ = 0.1, 0.2), the spread of the chromosome is not favored. Further, if this probability for the ribozymes approaches or equals to that for the chromosome (e.g., *P*_*LRTT*_ = 0.5, 0.9), the ribozymes would spread instead of the chromosome. This scenario is similar to the situation concerning the co-spread of ribozymes that we described in a previous study [7]. Finally, the spread of the chromosome is clearly favored by the increase of *F*_*IB*_ (Figure 4B, bottom-right panel), which enhances our argument on the role of self-cleavage. Such extensive analysis was also applied for all the other parameters, and the results show the influence of these parameters on the spread of the chromosome (in the Additional file 1: Figures S2-S5).

## Discussion

The results clearly show that an RNA chromosome can spread when it adopts a circular form and its “sense strand” is readily broken at the sites between genes (Figures 2, 3 and Additional file 1: Figure S1). Both the circularity and the inter-gene breaking are important for the spread of the chromosome (Figure 4). Therefore, the computer simulation study supports our hypothesis that circularity plus self-cleavage may have been used as a strategy for the emergence of a chromosome in RNA-based protocells (Figure 1).

In our model, we assumed that there was end-degradation for a linear RNA chain. The results show that a circular RNA chromosome, which can avoid end-degradation, would spread, whereas a “fictive” linear RNA chromosome with all the other properties which have been assumed for the circular chromosome (the high probability of turning into template and the self-cleaving feature) cannot spread (Figure 4A, top-left panel). This finding means that such an assumption is important for the scenario described here. However, is such an assumption conceivable?

In modern prokaryotes, circularity of the DNA chromosome is believed to be a strategy that is used to resist end-degradation, which is caused mainly by exonuclease cleavage of terminal phosphodiester bonds (of course, the circularity is also believed to be important as a strategy to prevent the chromosome-shortening in replication due to the 5′-3′ direction of DNA polymerization requiring RNA primers). RNA degradation in modern cells has been studied in detail [21], in which it was shown that exonuclease activities are apparently more prevalent than endonuclease activities. These clues imply that there may be some chemical reasons that rend the breaking of terminal phosphodiester bonds easier. Chemical RNA degradation in the absent of proteins was explored very early [22], however, as far as we know, to date there is no direct evidence showing that the breaking of terminal phosphodiester bonds is apparently easier than those in the middle. On the other hand, this assertion seems reasonable according to our knowledge in this area. The stability of a phosphodiester bond may be affected by the geometry of the linkage, where the position of the attacking 2′-oxygen nucleophile relative to the 5′-oxyanion leaving group is important [23]. Base stacking within a double stranded RNA can stabilize phosphodiester bonds by preventing the formation of the linkage conformation favoring the hydrolytic reaction. Likewise, base stacking within a single stranded RNA would also contribute to the chemical stability [22]. It may be expected that terminal base stacking would be less stable than base stacking within the chain, thus rendering the terminal phosphodiester bonds easier to break. Alternatively, one may speculate that the reason for the prevalence of exonucleases in modern cells is that the terminal phosphodiester bonds would be more exposed to possible enzymes in solution than internal ones. Then, likewise, they would also be more exposed to other possible catalysts. Indeed, it has been reported that some putative prebiotic oligopeptides (those that existed before the emergence of the translation mechanism) could catalyze the cleavage of RNA chains [24], albeit in these cases it was internal bonds that were cleaved. Therefore, in the RNA world, the breaking (or cleavage) of terminal phosphodiester bonds may have also been a significant issue.

Another kind of end-degradation of RNA may be the result of the spontaneous decay of nucleotide residues at the ends to their precursors. In modern cells, this kind of RNA degradation may be negligible because of the efficient nuclease activities that cleave phosphodiester bonds. However, in prebiotic conditions this effect may have been innegligible. In the scenario described in our model, nucleotides may decay to their precursors (with the probability *P*_*ND*_). It is unreasonable to assume that RNA cannot decay into nucleotide precursors until every phosphodiester bond has been hydrolyzed. At least, end-residues, which are more exposed to the solution may, like mononucleotides, be subject to decay, although to a less extent. Therefore, while we assumed that residues within an RNA chain cannot decay, we also assumed that end-residues may decay to nucleotide precursors, but with a smaller probability than that of free nucleotides (i.e., *P*_*NDE*_ should be smaller than *P*_*ND*_). The spontaneous decay of end-residues may result in the RNA end-degradation. A detailed mechanism that can be imagined is that the glycosidic bond between the ribose and the base of an end-residue may become exposed to solution and break, resulting in the dropping off of the base; then, without the protection of base stacking [22], the end phosphodiester bond between the ribose that is left and the second nucleotide residue may be easier to break, resulting in the loss of the ribose. Interestingly, an experimental study suggested that in some possible prebiotic conditions (e.g., in solution with relatively low pH), the glycosidic bond would be much more unstable than the phosphoester bond in a nucleotide [25]. Apparently, in these conditions, the spontaneous decay of end-residues cannot be neglected, and it may even be significantly more intensive than the breaking of internal phosphodiester bonds of RNA chains (i.e., *P*_*NDE*_ would be greater than *P*_*BB*_).

In the model, we only assumed the spontaneous decay of end-residues, but did not assume the possibly easier breaking of the terminal phosphodiester bonds of RNA chains. This is a conservative consideration. If both kinds of end-degradation exist, the benefit of circularity to prevent end-degradation can be expected to be more apparent.

The simulations per se do not demonstrate the de novo emergence of a chromosome from unlinked genes. The appearance of the first chromosome molecule would have been a rather occasional event, involving random chain ligation/recombination plus cyclization. Indeed, this single event may have occurred but subsequently been “abandoned” more than once, considering the high chance of RNA degradation. The real important issue is how the initial chromosome molecules could have had any chance of spreading, as was explored here. About the history of this transition, there are also some messages in the study. When ribozymes were simple in structure, and thus not very efficient in catalysis, they may have acted as good templates themselves (with higher *P*_*LRTT*_, e.g., 0.9 or 0.5, as shown in Figure 4B, bottom-left panel), and there would have been a world of these unlinked genes (see also our previous work [7], in which cooperation as well as competition of these ribozymes as unlinked genes was discussed). When the ribozymes evolved to a more efficient form with a more complicated structure, they may no longer be able to act as good templates (with lower *P*_*LRTT*_, e.g., from 0.2 to 0.01, as shown in Figure 4B, bottom-left panel), then the chromosome would have an opportunity to emerge (provided that it adopted a strategy of circularity and self-cleavage).

The simulation reported here was based on a model with a resolution at the monomer level, that is, individual nucleotides (A, U, G and C) and amphiphiles, and therefore is very computer-intensive. For simplification, the model adopts a two-dimensional grid system, like the traditional stochastic cellular automaton used by the replicator models [3-6,9]. However, here a grid room can accommodate a quantity of molecules that are deemed to be adjacent enough to interact with each other, which is different from the traditional stochastic cellular automation, in which one molecule occupies one grid room and molecular interactions occur between neighboring grid rooms. This treatment, somewhat similar to the approach used in a recent simulation study on prebiotic sequence evolution [26], saves computational costs and favors simulations involving complicated interactions at the monomer level. For simplification, the characteristic domains of the ribozymes that are assumed in the model are shorter (8 nt in the cases shown here, and 10 nt in some other cases) than in reality, and no structural features are considered. However, the principle that function is determined ultimately by sequence should have been sufficiently represented. Additionally, a self-cleaving site, which should be labeled by a hammerhead ribozyme subsequence, is only represented here by two residues (i.e., “U-G” in the cases shown here, see the legend to Figure 2). However, the mechanism of self-cleavage between genes should have been sufficiently represented. Certainly, increasing the length of the ribozymes and the self-cleaving label sites may bring our simulations more towards reality, but the system scale (represented, for example, by *T*_*NPPB*_) would increase correspondingly, and computation would become more cumbersome, even unmanageable.

In the simulation, it can be observed that the spread of the chromosome depends on the function of the genes that it carried. The control, similar to the chromosome but with a different sequence without any genes, cannot spread (white triangles in Figure 2 and in the Additional file1: Figure S1; white bars in Figure 3-top-row; grey bars in Figure 4B and in the Additional file 1: Figures S2-S5). When the function of the Rep becomes less efficient (in the Additional file 1: Figure S2, *P*_*TLR*_ decreases), or relatively less efficient compared with the non-enzymatic reaction (in the Additional file 1: Figure S2, *P*_*TL*_ increases), the spread of the chromosome is disfavored. Similar results are also shown for Nsr and Npsr (in the Additional file 1: Figure S2, *P*_*NFR*_ and *P*_*NF*_; *P*_*NPFR*_ and *P*_*NPF*_). These results emphasize that the spread of the chromosome depends on the ribozymes it encodes. However, for Asr, the result is somewhat different: the spread of the chromosome is disfavored when *P*_*AFR*_ decreases from 0.9 to 0.2 (in the Additional file 1: Figure S2), similar to the results for the other ribozymes; but in contrast, the spread is favored when *P*_*AFR*_ decreases from 0.2 to 0.01. Additionally, the spread is favored when Asr becomes less efficient in comparison with the non-enzymatic reaction (in the Additional file 1: Figure S2, *P*_*AF*_ increases). This difference should be caused by another factor that affects the spread of the chromosome. That is, a more efficient Asr would result in a faster membrane growth, and lead to protocell division, which at this early stage would be caused by random physical forces in the environment as the protocells increased in size. As a result, the ribozymes (Rep, Nsr, Npsr and Asr) are more likely to separate from the chromosome accompanying the protocell division, and will not “serve” the chromosome any more. The result that a higher probability of protocell division disfavors the spread of the chromosome (in the Additional file 1: Figure S3, *P*_*CD*_) supports this argument.

This result, concerning *P*_*CD*_, is a little surprising per se. In our previous study on the cooperation of different ribozymes without the existence of a chromosome, we showed that the co-spread of the ribozymes was disfavored when *P*_*CD*_ was higher [7]. The reason is that a higher rate of protocell division may result in more intensive gene loss during the division. Before the present simulation was conducted, we had expected that the spread of the chromosome (with linked genes) might resist faster cell division apparently.

Noticeably, the numbers of ribozymes in the system are quite few compared with the number of the chromosome (Figure 2 and Figure 3-top-right; see also Figure 4B and in the Additional file 1: Figures S2-S5), except for the cases in which the chromosome cannot spread and ribozymes become prosperous when the probability of ribozymes acting as template themselves rises (Figure 4B, *P*_*LRTT*_). In the primordial strategy suggested here, the ribozymes are only byproducts of the chromosome replication (via self-cleaving, see Figure 1). As a result, the numbers of ribozymes are only retained at a low level. In this situation, fast cell division would be clearly deleterious. Ribozymes produced from the chromosome are likely to “serve” the chromosome only for a short time, and protocells with the chromosome might lack ribozymes of this kind or that kind. This phenomenon might be called “ribozyme loss”.

The emergence of the chromosome in RNA-based protocells would favor the appearance of more genes and the corresponding ribozymes and there would not be the problem of gene loss accompanying the protocell division. The problem of “ribozyme loss” is not as serious as that of gene loss, because the genes are always preserved in the chromosome and the ribozymes would be produced continuously from the chromosome. Subsequently, of course, a mechanism of transcription that could use tags like promoters in modern cells may have emerged to produce more copies of ribozymes, thereby, alleviating the problem of ribozyme loss. Further study to model this possible subsequent stage will be important and interesting, particularly to show to what extent of complexity the RNA world may have developed before the advent of DNA and proteins.

## Methods

An *N* × *N* grid was used for the system, with toroidal topology to avoid edge effects. The objects in the model are nucleotide precursors’ precursors, nucleotide precursors, nucleotides (in different types, A, U, G and C), RNA, amphiphile precursors, amphiphiles, and protocells. Only molecules within the same “grid room” can interact. Membrane may assemble (from amphiphiles) at the edge of a grid room and then the grid room is occupied by a protocell. When a protocell moves to an adjacent naked grid room, the protocell would push away molecules in that room. When a protocell divides, amphiphiles on the membrane and molecules in the protocells would be distributed randomly between the two offspring protocells. One of the offspring protocells would occupy an adjacent naked grid room and push away molecules in that room. Only protocells at adjacent grid rooms can fuse to each other.

An RNA containing a characteristic sequence domain (presumed arbitrarily) may function as a corresponding ribozyme: Rep, Npsr, Nsr, or Asr. However, the RNA should be shorter than 1.5 times of the characteristic domain; otherwise, it is deemed that the “correct” structure would be interfered by the redundant residues and it would not act as the ribozyme. The sense chain of the chromosome has a sequence of these ribozyme domains in a tandem and circular way. The antisense chain of the chromosome is complementary to the sense chain.

**Figure 5:**
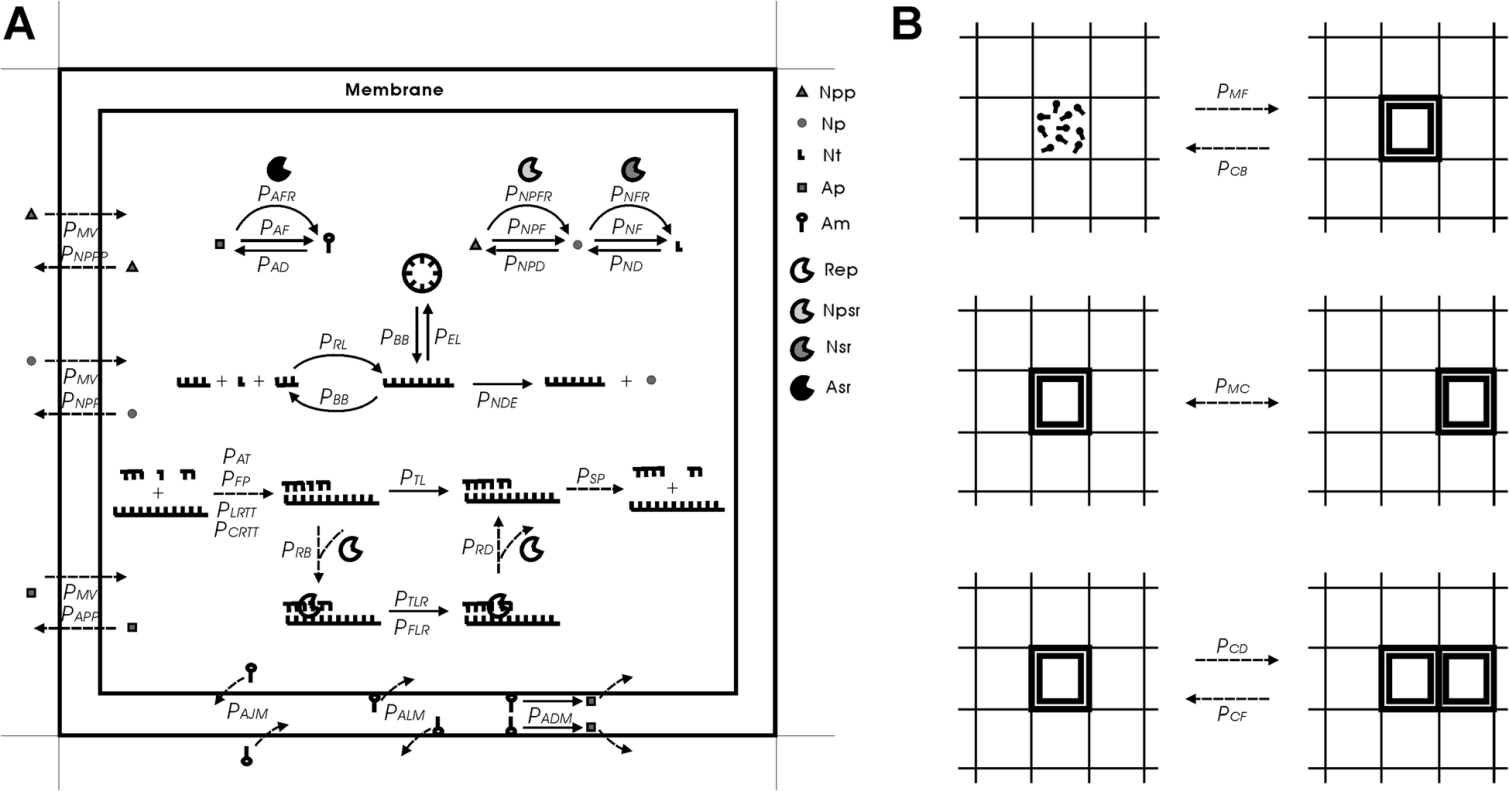
**Events occurring in the model and their associated probabilities.** Solid arrows represent chemical events and dashed arrows represent other events. **(A)** Events occurring in a grid room. Legends: Npp, nucleotide precursor’s precursor; Np, nucleotide precursor; Nt, nucleotide (A, U, C, or G); Ap, amphiphile precursor; Am, amphiphile; the notations of ribozymes are the same as in the text. Note that for the template-directed synthesis, the probability of a circular RNA turning into a template would be *P*_*CRTT*_ instead of *P*_*LRTT*_ and the synthesis may start at a random site, whereas other associated events are the same as those for the linear RNA (shown in the figure). Some factors associated with the events are explained in the text. One of these factors, *F*_*IB*_ , is important for the topic of the present study. For a particular intermediate site between two genes in a linear RNA (see the legend to Figure 2 for details of the assumption concerning the site), the probability of phosphodiester bond breaking (*P*_*BB*_) is increased by multiplying the factor, *F*_*IB*_, which represents the consideration of the self-cleaving effect. **(B)** Events concerning the behaviors of the protocells. 9 grid rooms are shown in each panel.

In a simulation case, nucleotide precursors’ precursors in the quantity of *T*_*NPPB*_ and amphiphile precursors in the quantity of *T*_*APB*_ were introduced at the initial step, some empty protocells were inoculated soon after the initial step, and then some protocells containing a few molecules of the ribozymes (Rep, Npsr, Nsr and Asr) and the circular chromosome with linked genes were inoculated some steps later. “Internal” events (each with a probability in a time step, Table 1) in the model govern the whole dynamic process occurring in the system (Figure 5), step by step. Besides the probabilities, there are a few other parameters in the model (Table 1). The setting of values of the probabilities should obey some logics according to their relationship. Additionally, there are some detailed assumptions considering real situations associated with some of the events. A more detailed description of the events in the model and these associated considerations is provided in the following.

### Events occurring in a time step and associated parameters

A nucleotide precursor’s precursor may transform to a nucleotide precursor, with the probability *P*_*NPF*_ (see Table 1 for a description of the abbreviation and those appearing below; also refer to Figure 5) in a non-enzymatic way, or with *P*_*NPFR*_ when catalyzed by an Npsr. A nucleotide precursor may transform to a nucleotide (randomly as A, U, C, or G) with *P*_*NF*_ in a non-enzymatic way, or with *P*_*NFR*_ when catalyzed by an Nsr. A nucleotide may decay into a nucleotide precursor with *P*_*ND*_. A nucleotide residue at the end of an RNA chain may decay into a nucleotide precursor with *P*_*NDE*_, A nucleotide precursor may decay into a nucleotide precursor’s precursor with *P*_*NPD*_. Two nucleotides or RNA molecules may be ligated with *P*_*RL*_, forming longer chains. An RNA molecule may be ligated end-to-end with *P*_*EL*_, forming a circular chain. A phosphodiester bond within an RNA chain may break with *P*_*BB*_. For a particular intermediate site between two genes (see the legend to Figure 2 for a detailed explanation) in a linear RNA, *P*_*BB*_ is multiplied by a factor, *F*_*IB*_ (>1), which represents the consideration of the self-cleaving effect. A linear RNA may turn into a template (unfolding) with *P*_*LRTT*_, whereas a circular RNA may turn into a template with *P*_*CRTT*_. A template may attract nucleotides or oligomers with *P*_*AT*_ by base-pairing (the probability of false base-pairing tolerated at each residue site is *P*_*FP*_). Nucleotides and oligomers aligned adjacently on the RNA template may be ligated to each other with *P*_*TL*_ (template-directed ligation) in a non-enzymatic way. A Rep molecule may bind onto an RNA template with *P*_*RB*_ and drop from the template with *P*_*RD*_. If there is a Rep on the template, the template-directed ligation may occur with *P*_*TLR*_; however, if one or both base pairs flanking the ligation site are false, the ligation would not occur unless another probability, *P*_*FLR*_, is satisfied. The substrates or the products on the RNA template may dissociate if base pairs between them can separate (each base pair may separate with *P*_*SP*_).

An amphiphile precursor may transform to an amphiphile with *P*_*AF*_ in a non-enzymatic way, or with *P*_*AFR*_ when catalyzed by an Asr. Amphiphiles (with a lower limit of quantity *L*_*AM*_) may assemble into a membrane at the edge of a grid room with *P*_*MF*_, encompassing molecules within it and forming a “protocell”. A free amphiphile may decay into an amphiphile precursor with *P*_*AD*_, whereas an amphiphile within a protocell membrane (not within the protocell but only on the membrane) may decay with *P*_*ADM*_. A free amphiphile may join a protocell’s membrane with *P*_*AJM*_*,* whereas an amphiphile within a protocell membrane may leave it with *P*_*ALM*_. Nucleotides and RNA are assumed to be impermeable, whereas a nucleotide precursor’s precursor, a nucleotide precursor and an amphiphile precursor may diffuse across the membrane with *P*_*NPPP*_, *P*_*NPP*_ and *P*_*APP*_, respectively. A protocell may divide into two with *P*_*CD*_ and two adjacent protocells may fuse into one with *P*_*CF*_. A protocell may break (into free amphiphiles) with *P*_*CB*_. The probabilities of the decay of a nucleotide precursor (*P*_*NPD*_), a nucleotide (*P*_*ND*_), a nucleotide residue at the end of an RNA (*P*_*NDE*_), an amphiphile (*P*_*AD*_), and the probability of phosphodiester bond breaking in an RNA chain (*P*_*BB*_), are multiplied by a factor, *F*_*DO*_ (>1), when these events occur out of protocells. This represents the consideration that water activity should be higher outside the protocells.

Before the next time step, molecules and protocells in a grid room may move into adjacent rooms. A nucleotide, a nucleotide precursor, a nucleotide precursor’s precursor, an amphiphile, or an amphiphile precursor may move with *P*_*MV*_, whereas a protocell may move with *P*_*MC*_.

### Logical setting of the probabilities according to their relationship

Ribozymatic reactions should be much more efficient than corresponding non-enzymatic reactions, so *P*_*TLR*_> > *P*_*TL*_, *P*_*NPFR*_> > *P*_*NPF*_, *P*_*NFR*_> > *P*_*NF*_, and *P*_*AFR*_> > *P*_*AF*_. “Template-directed ligation” should be significantly more efficient than “random ligation”, so *P*_*TL*_> > *P*_*RL*_. The end-to-end ligation of an RNA chain (cyclization) should be similar in efficiency to the random ligation of two different RNA chains, so *P*_*EL*_ should be of the same order as *P*_*RL*_. Nucleotide residues in an RNA chain should be protected. Here, nucleotide residues within the chain are assumed to be unable to decay, whereas those at the end of the chain decay at a rate lower than that of free nucleotides, i.e., *P*_*NDE*_ < *P*_*ND*_. Amphiphiles within a membrane should be protected, so *P*_*ADM*_ < *P*_*AD*_. Because of the self-assembly feature of amphiphilic molecules, *P*_*MF*_> > *P*_*CB*_ and *P*_*AJM*_> > *P*_*ALM*_. The movement of molecules should be easier than protocells, so *P*_*MV*_ > *P*_*MC*_. A nucleotide precursor’s precursor should be easier to permeate through the membrane than a nucleotide precursor, so *P*_*NPPP*_ > *P*_*NPP*_. Other considerations may include: *P*_*BB*_ may be of the same order as *P*_*RL*_, *P*_*ND*_ ≥ *P*_*NF*_, *P*_*NPD*_ ≥ *P*_*NPF*_, *P*_*AD*_ ≥ *P*_*AF*_, and *P*_*APP*_ ≥ *P*_*NPP*_.

### Some detailed assumptions considering real situations

The probability of the separation of the two strands of a duplex RNA is assumed to be *P*_*SP*_^*q*^, wherein *q* equals to *r*^1/2^, and *r* is the number of base pairs in the duplex. When *r* increases and thus *q* increases, *P*_*SP*_^*q*^ would decrease (because *P*_*SP*,_ as a probability, has a value between 0 and 1). That is, the separation of the two strands would be more difficult if the base pairs are more. The introduction of the square root (i.e., *q* equals to *r*^1/2^) represents the consideration of the synergistic effect of the separation of the base pairs (if a base pair is separated, the base pairs flanking it would be easier to separate).

The probability of membrane formation is assumed to be 1-(1-*P*_*MF*_)^x^, where x equals to *a*-*L*_*AM*_ + 1, and *a* is the number of amphiphiles in the grid room. When *a* equals to *L*_*AM*_, the probability of membrane formation equals to *P*_*MF*_. This assumption concerns the consideration that the more amphiphiles there are, the more possible it is that they would assemble to form a cell membrane.

The probability of an amphiphile leaving the membrane is assumed to be *P*_*ALM*_ / [1 + *F*_*OP*_ × *n*/(*b*/2)^3/2^], where *n* is the quantity of inner impermeable ions, including nucleotides and RNA (measured by the number of nucleotide residues), and *b* is the quantity of amphiphiles within the membrane. Wherein, *b*/2 (there are two layers in the membrane) is a “scale” representation of the surface area of the membrane. Consequently, (*b*/2)^3/2^ is a scale representation of the cellular space. Thus, *n*/(*b*/2)^3/2^ is a representation of the concentration of the ions. *F*_*OP*_ × *n*/(*b*/2)^3/2^ represents the consideration for the “osmotic pressure effect”; a higher concentration of the inner impermeable ions would cause the protocell to be more swollen, and thus amphiphiles within the membrane are less likely to leave [27].

The probability of a nucleotide precursor permeating into a protocell is assumed to be [1-(1-*P*_*NPP*_)^y^] / [1 + *F*_*DE*_ × *n*/(*b*/2)^3/2^], where *n* is the quantity of inner impermeable ions and *b* is the quantity of amphiphiles within the membrane (Note that, here *F*_*DE*_ corresponds to *F*_*SI*_ in our previous work [7,20], and this assumption formula has been modified a little to more closely approach the real situation). The index y equals to (*b*/*L*_*AM*_)^3/2^, which represents the consideration of the limiting effect of the cellular space on the influx of nucleotide precursors. When *b* equals to *L*_*AM*_ (the lower limit of the number of amphiphiles to form a protocell membrane), y equals to 1. When the *b* increases, meaning that the cellular space increases correspondingly, the probability of a nucleotide precursor permeating into the protocell would become greater. *F*_*DE*_ × *n*/(*b*/2)^3/2^ represents the consideration of the effect of Donnan’s equilibrium [28]. See Ref. [20] for a detailed explanation of the influence of Donnan’s equilibrium on the RNA-based protocell. Similarly, the probability of a nucleotide precursor’s precursor permeating into a protocell is assumed to be [1-(1-*P*_*NPPP*_)^y^] / [1 + *F*_*DE*_ × *n*/(*b*/2)^3/2^]. The probability of an amphiphile precursor permeating into a protocell is assumed to be 1-(1-*P*_*APP*_)^y^, wherein the effect of Donnan’s equilibrium is not considered because amphiphile precursors are assumed to be uncharged molecules.

The probability of protocell division is assumed to be *P*_*CD*_ × (1-2 × *L*_*AM*_/*b*), where *b* is the quantity of amphiphiles within the membrane. When *b* is no more than twice that of *L*_*AM*_ (the lower limit of the number of amphiphiles to form a protocell membrane), the probability is no more than 0, i.e., the protocell could not divide. This assumption represents the consideration that the larger the protocell, the more the probability that it would divide.

The probability of the movement of an RNA molecule is assumed to be *P*_*MV*_ / *m*^1/3^, where *m* is the mass of the RNA, relative to a nucleotide. This assumption represents the consideration of the effect of the molecular size on the molecular movement. The molecular diffusion is assumed to be affected in a one-dimension scale, thus the cubic root of the mass (as a three-dimension factor, comparable to volume of the molecule), was used here.

(**Note:** The source code of the simulation program, written in C language, can be obtained from the corresponding author on request by e-mail. The source code could help readers to understand the model better if they have sufficient background knowledge in programming. At least, the readers can repeat our simulation by running the program themselves – even more, they may adjust the parameter values to address the issues of interest to them).

## Conclusions

Early life should be simple but capable of Darwinian evolution. This logic breeds the suggestion of an RNA world as an early stage of life because RNA, as one sort of molecules, can play dual role as genetic materials and functional materials (thus making Darwinian evolution possible). Since one molecule can only carry limited functions (usually only one), the cooperation of different functional RNA molecules within a “protocell” seems inevitable in the evolution towards higher efficiency. However, the division of such a protocell would risk losing some of these RNA molecules unless they could be linked, as a chromosome.

“Unfortunately”, there would be some apparent difficulties for the emergence of the chromosome. Inspired by features of viroids, we suppose that these difficulties could have been overcome if circularity plus self-cleavage was adopted as a strategy by the RNA chromosome. Via a computer simulation, we show that this strategy is plausible, and both circularity and self-cleavage are important for the spread of the chromosome in the model system. The strategy is consistent with the characteristic of simplicity for early life.

The emergence of a chromosome would be a breakthrough for the evolution towards complicated RNA-based protocells with many genes. In a more general sense, this event, entailing a membrane-bounded system with a central genetic molecule composed of linked genes, may represent a crucial step towards “life in a full sense” – the one capable of undergoing “unlimited” Darwinian evolution, considering that this cell-like system might evolve towards complexity/efficiency “endlessly” accompanying the introduction of different genes with various functions.

## Reviewers’ comments

We are grateful to the reviewers for their thoughtful analysis and critique of our manuscript. We think that this manuscript, which addresses an interesting but certainly debatable problem in the origin of life, is very suitable to appear in this journal, which has a policy to publish reviewers’ comments and authors’ responses together with a manuscript. In our response to the reviewers below, we have omitted some minor points brought to our attention (language, additional references, formatting, etc.), correcting them directly in the manuscript instead.

### Reviewer 1: Dr. Sergei Kazakov, Somagen Inc, Santa Cruz, USA (nominated by Dr. Laura Landweber, Princeton University, USA)

#### *Reviewer comments*

The authors open their paper with the following sentence “It is now popularly believed that an RNA world existed in early evolution.”

**– –** I would not consider an RNA world as a belief but rather as a feasible hypothesis, based on the known ability of RNA molecules to perform multiple functions that are performed by DNA, RNA and proteins in modern biology.

#### *Authors’ response*

Yes, the idea of the RNA world is a hypothesis, rather than a belief. What we mean here is that this hypothesis is now supported by quite a lot of evidence so that it is widely accepted in the field of the origin of life. To avoid misunderstanding, we have changed the word “believed” to “accepted” in the revised version. Thanks.

#### *Reviewer comments*

The authors focus on one of several missing links in the RNA World hypothesis, namely on the emergence of an RNA “chromosome carrying linked genes” that encode ribozymes. The authors state that such “chromosome would be much longer than individual genes, and thus more likely to degrade and less likely to replicate completely; the copying of the chromosome might start at middle sites and be only partial; and, … the synthesis of distinct ribozymes [genes] would become problematic.” Furthermore it is proposed that “inspired by features of viroids, which have been suggested as living fossils of the RNA world… these difficulties could have been overcome if the chromosome adopted a circular form and small, self-cleaving ribozymes (e.g. hammerhead ribozymes) resided at the sites between genes.”

#### *Authors’ response*

Yes, this is just the central idea of our paper. The reviewer’s description is quite right, thanks.

#### *Reviewer comments*

The idea about the emergence of circular RNAs in RNA World is reasonable but it is not novel. For example, we state that “Circular RNAs have several features that could have been useful at an early stage of evolution: (a) they remain in one piece after a single random cleavage event, allowing recovery of their structure through re-ligation of their ends; (b) they can serve as a template for rolling circle replication and amplification; and (c) they have fewer conformational degrees of freedom compared to their linear counterparts, and therefore can bind to other molecules (or fold) with less entropic cost” [29].

#### *Authors’ response*

Yes, the emergence of circular RNAs in the RNA world may have other advantages, like those stated in the work coming from the reviewer’s group [29]. The novelty of the idea here is that the circularity may be adopted (along with the self-cleavage) as a strategy to overcome those difficulties of the emergence of an RNA chromosome in the RNA world. Any other possible advantages of the circularity would “increase the likelihood” of the adoption of such a strategy. Interestingly, just in this work [29], the reviewer and his coworkers indicated (by experiments) the condition that may have favored the circularization of RNA – freezing (and dehydration). Indeed, as explained by them, the RNA world may have been characterized by a freezing circumstance, “in which RNA was relatively protected against degradation”.

#### *Reviewer comments*

In this manuscript, Ma et al. offered three novel features (or factors) supporting the emergence of circular RNAs:

“First, by circularity, the chromosome could evade the degradation starting from its chain ends, and this would compensate to some degree the disadvantage concerning degradation due to its longer chain. The end-degradation may be caused by the easier breaking of terminal phosphodiester bonds than internal ones, or by the spontaneous decay of terminal nucleotide residues. If such end-degradation was strong, such compensation may be expected to be significant.”

**– –** The proposed “end-degradation” factor is purely speculative and is not based on known properties of RNA. Rather, it is feasible that RNA degradation under prebiotic conditions, in the absence of nucleases, was catalysed by low or high pH as well as by metal ions in combination with high temperatures; none of these conditions lead to preferential cleavage of the terminal rather than internal internucleotide bonds in polynucleotides (see e.g. [30] and references therein). Internal cleavages, however, would compromise the circularity of the chromosome, and it is necessary to consider the occurrence of ligating enzymes that can re-ligate the cleavage to restore the circular form of RNA chromosomes.

#### *Authors’ response*

Yes, we have discussed this issue in detail in the Discussion section – indeed, “as far as we know, to date there is no direct evidence showing that the breaking of terminal phosphodiester bonds is apparently easier than those in the middle. On the other hand, this assertion seems reasonable according to our knowledge in this area”. Even if no preferential cleavage of the terminal bonds exists, there could be other mechanisms for the “end-degradation”, for example (as discussed in the text), (1) the more exposure of the terminal phosphodiester bonds to some possible prebiotic peptides that cleaved RNA [24], (2) the spontaneous decay of nucleotide residues at the ends. In fact, in the model used here for computer simulation, “we only assumed the spontaneous decay of end-residues” as the mechanism of the end-degradation, which is, apparently, “a conservative consideration”.

As to “the occurrence of ligating enzymes that can re-ligate the cleavage to restore the circular form of RNA chromosomes”, mentioned by the reviewer, we think that the idea is interesting and could be considered in our future study, but is perhaps beyond the scope of our topic here.

#### *Reviewer comments*

“Second, the circular topology is expected to further impede (sterically hinder) the folding of distinct ribozyme domains in the chromosome, making the chromosome more suitable to act as a template in the replication … because of the interference of individual ‘ribozyme domains’ with each other. However, the effect of this factor may be limited (and perhaps sequence-dependent).”

**– –** This factor is feasible, but it definitely would limit the sequence diversity of these RNA chromosomes.

#### *Authors’ response*

Yes, the logic is: (1) an RNA chromosome (even a linear one) with linked “ribozyme domains” was expected to be a better template because of the interference of the domains with each other (concerning base-pairing), preventing the formation of tight structures as in free ribozymes; (2) however, the interference may be limited, depending on the RNA sequence of the ribozymes; (3) a circular topology is expected to further impede (sterically hinder) the folding of distinct ribozyme domains in the chromosome, making the chromosome more suitable to act as a template in the replication.

#### *Reviewer comments*

“Third, there would be no issue concerning the starting point of copying” from a large circular RNA chromosome template.

**– –** It is true that a circular template would allow copying its entire sequence independently of the starting point through rolling-circle-amplification (RCA). It is a valid and good point.

#### *Authors’ response*

Yes, thanks; but note that the replication mechanism assumed in the present model is not that of RCA.

#### *Reviewer comments*

“Fourth …if the ‘sense strand’ of the chromosome tends to be broken at the sites between genes, corresponding ribozymes may be produced this way. A hammerhead ribozyme catalyzing self-cleavage may have existed at these sites.”

**– –** Hammerhead is a very effective ribozyme and with high probability would compromise the circularity of such RNA chromosomes by cleaving them. It is more likely that only smaller, less effective ribozymes could realistically be considered as part of circular chromosomes. Moreover, the consensus sequence of the in-cis cleaving hammerhead ribozyme is about 30 nt long, and the author’s computer modeling limits the length of the entire chromosome to this size. The latter demonstrates a serious problem with the author’s approach, since the model does not include longer chromosomes, which may behave differently from the small ones.

#### *Authors’ response*

Firstly, perhaps there is a misunderstanding here. Yes, hammerhead is a very effective ribozyme, but they would only work when the circularity is break, as mentioned in the second paragraph of the Results section: “It should be noted that the self-cleaving effect would not occur in the circular chromosome … This is consistent with the findings in viroids, wherein the embedded hammerhead ribozyme is, in fact, inactive in the circular RNA chromosome, but would work during the rolling-circle replication, when it resides in the long linear RNA, between the genome copies [11,12].” A similar notion has also been mentioned in the legend of Figure 1.

Secondly, indeed, the lengths of the self-cleaving ribozyme and the RNA chromosome assumed in the simulation are much shorter than those that were possible in reality. However, such a case is somewhat a commonplace for computer simulation. In a computer simulation, sometimes simplifications or unrealistic assumptions have to be adopted in order to show the underlying mechanisms in a clear mode or even, as in this case, merely to avoid cumbersome computation. The key issue is that to what extent that the simplifications or assumptions may affect the results, influencing the relevance of the simulation to the realistic situation. It should also be admitted that we cannot say with certain that the behavior difference mentioned by the reviewer would not exist. Nonetheless, we should take an open mind at cases like this, appreciating the valuable message transferred by the simulation. For example, in traditional, popularly used “replicator” models in the field of the origin of life (e.g. those summarized in [6] and that used in [9]), simplifications and unrealistic assumptions are much more apparent, but we can learn a lot from them.

Further discussion about the length of the self-cleaving ribozyme and that of the RNA chromosome will be found below, where the reviewer comments them in other respects.

#### *Reviewer comments*

The authors describe the hammerhead ribozyme as “the smallest ribozyme found in nature”.

**– –** This is not true, since the smallest ribozyme motif is just 7-nt long ([31], and references therein). In fact, enzymes of such smaller size would better suit the authors “circular RNA chromosome” hypothesis.

#### *Authors’ response*

Yes, the hammerhead ribozyme is not the smallest ribozyme we know. As the reviewer mentioned, the active motif of the smallest ribozyme we know is only 7-nt long [31]. However, it is an artificial, pared-down version of a 31-nt long RNA, which itself is excised from the 5′ end of the rRNA intron of *Tetrahymena*. To make our meaning clearer, we have changed the word “nature” to “living beings”.

The 31-nt RNA is also a self-cleaving RNA, and similar to the hammerhead ribozyme in size. Interestingly, its pared-down version – the 7-nt motif can still perform the self-cleaving reaction [32]. Therefore, it is indeed possible, as the reviewer said, smaller, less effective ribozymes may have played the hammerhead ribozyme’s role that is supposed in our hypothesis, especially considering that a preliminary RNA chromosome cannot be very long. Thanks to the reviewer for this comment. Because the comment and our response would appear together with the manuscript, we have left unchanged our statement in the text about the hammerhead ribozyme as the most plausible candidates of the self-cleaving RNA motif in our hypothesis.

#### *Reviewer comments*

In order to determine the plausibility of their hypothesis, the authors conducted a computer simulation using a Monte-Carlo method.

**– –** As mentioned above, I am concerned that the authors are using some erroneous parameters/factors for the simulation input. Furthermore, taking in account only short chromosomes is not sufficient to test the author’s original hypothesis describing RNA “chromosome [that] would be much longer than individual [ribozyme] genes”.

Larger RNA chromosomes may represent a challenge not only for the modeling. The authors may consider that other arguments may argue against the existence of larger RNA chromosomes. For example, the dissociation between sense/template and antisense/transcript RNA strands would be hampered due to a high thermostability of RNA-RNA duplexes. Without strand dissociation, the ribozyme genes and self-cleaving units cannot fold into their active conformation. In fact, this is probably the strongest argument against the possible existence of large RNA chromosomes. The length limitation for circular RNAs lies probably at about 150 nt. RNA circles of this or shorter sizes could be amplified via RCA without the need for separate strand-displacement activity of RNA polymerase ribozymes. The 5′ end of the antisense/transcript strand would dissociate automatically because the duplex formed by a small circular template is stiff and does not allow the formation of a duplex longer than half of the length of the circle ([33], and references therein).

#### *Authors’ response*

Firstly, as to the parameters/factors for the simulation input, we cannot say that they are certainly right. Actually, computer simulation in the field of the origin of life can hardly take “right” parameters/factors, or even a “right” model, because we know too little about the process. Nevertheless, computer simulation is very useful in this field. Perhaps somewhat oddly, the reason is also that we know too little about the process. The simulation is expected to provide clues for us to understand the process, and in practice, to direct our explorations in laboratory. First, when setting the parameters/factors, we take into consideration those apparent logics and our previous, limited knowledge (in this work, see the description in the Method section, particularly in “Logical setting of the probabilities according to their relationship” and “Some detailed assumptions considering real situations”). Then, when we find the appearance of a phenomenon is sensitive to the setting of some parameters/factors, we judge that these parameters/factors may have been important for the mechanism of the phenomenon (just like those that were indicated in Figure 4). Finally, in contrast, the outcome is expected to be relatively robust against the variation of other parameters/factors (like those that were shown in Additional file 1: Figure S2-S5). Further discuss about the parameterization will appear below in our response to a comment from Reviewer 3.

Secondly, the meaning of “chromosome [that] would be much longer than individual [ribozyme] genes” should be understood as in respect of the relative length, e.g., in the model here, the chromosome is four times as long as individual ribozymes. Hence, the judgment “taking in account only short chromosomes is not sufficient to test the author’s original hypothesis” should have arisen from a misunderstanding.

Thirdly, in this work we do not intend to argue against the possibility of the existence of a long circular RNA chromosome. The reason why we do not introduce a long RNA chromosome is merely associated with the consideration of computational burden. However, the argument of the reviewer in this respect, particularly concerning the problem of strand dissociation, is interesting and seems reasonable. Indeed, below we will find another reasonable argument from Review 2: “the authors assume that circularization destabilizes the folding of RNA molecules and thereby renders them more suitable as templates. However, a circular RNA might behave nearly as a linear molecule (at least locally) if its length is sufficiently long”. In both the two opinions, it seems that if a preliminary circular RNA chromosome appeared in the RNA world, it may have been quite short, and cannot evolve to significant longer ones. If so, DNA may have arisen very early in the RNA world, serving as the carrier of genetic information -- for the appearance of more and larger genes. However, there may still be other issues. For example, DNA may be more suitable to act as template, but longer DNA chromosomes cannot yet escape the problem of strand dissociation, perhaps more serious because single chain DNA, unlike RNA, can hardly self-fold to prevent its annealing with its complement. If it is difficult to imagine a ribozyme that acted as a helicase, perhaps -- proteins emerged earlier (before DNA), helping with the strand dissociation? But how could proteins have emerged before the emergence of a large genome, especially considering the complexity of the translation mechanism? Certainly, that is already beyond the scope of our topic here.

#### *A further comment from this reviewer after our response*

In the response to one of my comments, the authors wrote “… note that the replication mechanism assumed in the present model is not that of RCA.” However, RCA is the only mechanism (in the absence of helicases) to generate single-stranded RNA amplicons that then could be cleaved by the encoded ribozymes. As I described in another comment: “For example, the dissociation between sense/template and antisense/transcript RNA strands would be hampered due to a high thermostability of RNA-RNA duplexes. Without strand dissociation, the ribozyme genes and self-cleaving units cannot fold into their active conformation”. So without including RCA or an alternative mechanism for template-transcript strand separation into the model, the ribozyme units would remain in dsRNA form and could not fold into active conformation.

#### *Our response to this comment*

Indeed, as the reviewer said: “RCA is the only mechanism (in the absence of helicases) to generate single-stranded RNA amplicons that then could be cleaved by the encoded ribozymes”. However, it seems that this mechanism is heavily depended upon the existence of an efficient RNA polymerase (e.g., for those viroids, it is a protein enzyme in host cells). We doubt if an efficient RNA polymerase ribozyme could exist in this early stage of the RNA world – instead, the RNA replicase ribozyme in the present model is a template-dependent ligase with a relatively low efficiency (please refer to one of our previous papers [18]). Perhaps RCA is a mechanism that evolved later, after the emergence of an efficient RNA polymerase (being it protein enzyme or ribozyme), and it is likely a strategy of parasites. Certainly, the result would also be interesting if an efficient RNA polymerase ribozyme and the RCA mechanism could be introduced into our model – however, these changes are not easy to make, and may form the topic of another study in future.

As to the dissociation of the RNA strands, there would be no serious problems if the circular RNA chromosome was short. As mentioned by the reviewer: “… the duplex formed by a small circular template is stiff and does not allow the formation of a duplex longer than half of the length of the circle [33]”. This point is reasonable and should be valid for the replication of the circular RNA chromosome even if the replication mechanism is not RCA. Certainly, when the chromosome becomes longer, the issue might become more complicated – perhaps relevant to the emergence of DNA and helicase (protein), as already mentioned above.

### Reviewer 2: Dr. Nobuto Takeuchi, National Center for Biotechnology Information, NIH, USA (nominated by Dr. Anthony Poole, Stockholm University, Sweden)

#### *Reviewer comments*

In their paper, Ma et al. investigate the origin of chromosome-like molecules in RNA-based protocell systems using computer simulations. The authors consider two key factors that seem relevant to the origin of such molecules: circular RNA and self-cleaving ribozymes (such as seen in viroids). The authors assume that circularization unfolds an RNA molecule and thereby renders it more suitable as a template; in addition, it removes the chain ends and so increases the resistance of an RNA molecule against hydrolytic (exo-) degradation. Moreover, the authors suggest that self-cleaving ribozymes can be used to implement a primitive form of “transcription” in a wide sense (i.e., the production of catalysts from templates) if such ribozymes intervene multiple other ribozymes that are concatenated with each other. Using computer simulations, the authors show that protocells containing such primordial chromosomes can survive if both circularization and sufficiently high self-cleavage activity are assumed.

Both the ideas of circularization and primitive transcription based on self-cleavage are novel and worthy of consideration in exploring the potentials of RNA-based evolvable systems. Particularly intriguing is the possibility that these ideas might be directly tested by experiments (at least in terms of their basic premises).

#### *Authors’ response*

Yes, the reviewer’s summary about our work is correct and accurate, thanks.

#### *Reviewer comments*

The authors’ analysis of their model, however, focuses only on the question of whether primitive chromosomes can survive when they are introduced into a model system. From a theoretical point of view, this leaves unanswered two crucial questions regarding the evolution of primitive chromosomes. First, exactly what selective advantage enables protocells containing chromosomes to out-compete those lacking chromosomes? Second, can primitive chromosomes emerge through evolution in a protocell system where chromosomes are originally absent?

Regarding the first question, it is important to note that primitive chromosomes have an intrinsic fitness disadvantage over unit ribozymes at the within-protocell level, as described in the Introduction section of the paper. In the authors’ model, chromosomes are four times longer than unit ribozymes, which leads to a four-fold disadvantage for chromosomes over unit ribozymes in terms of both replication and decay, amounting to a 16-fold fitness disadvantage. In a previous study, Maynard Smith and Szathmary [8] show that, under a few key assumptions, such an intrinsic disadvantage of chromosomes can be outweighed by the fitness advantage of chromosomes at the between-protocell level, namely, the reduction by chromosomes of assortment load. By contrast, the current study seems to indicate that chromosomes must have an extremely large additional advantage provided by the circular structure in order to survive in a protocell system. Specifically, the authors’ model assumes that chromosomes decay nearly one order of magnitude slower than unit ribozymes (*P*_*BB*_ < <*P*_*NDE*_) and function as templates nearly two order of magnitude more frequently than unit ribozymes (*P*_*CRTT*_> > *P*_*LRTT*_) on the basis of their circular structure. Taken together, circularization provides about a 1000-fold advantage for chromosomes, which apparently far outweighs the 16-fold disadvantage described above. Interestingly, the model shows that such an excess advantage is necessary for survival of primitive chromosomes (Figure 4). A possible interpretation of this result is that the reduction of assortment load caused by chromosomes provides only a negligible advantage. This interpretation, however, is at odd with the conclusion of Maynard Smith and Szathmary; moreover, it does not explain the fact that the advantage due to circularization must far exceeds the disadvantage due to a longer length. An alternative interpretation is that the primitive transcription based on self-cleavage incurs a large fitness cost to chromosomes, so that chromosomes need an extra fitness advantage to survive. This is a more plausible interpretation because self-cleavage entails self-destruction. To clarify the issues considered above, it is necessary to compare the advantages and disadvantages of primitive chromosomes and thereby to pinpoint exactly what enables primitive chromosomes to survive.

#### *Authors’ response*

To our surprise, here the reviewer showed his profound insights into the problem. Nonetheless, we have something to say. This is just the charm of the policy of publishing reviewers’ comments and authors’ responses together with a manuscript.

Firstly, we doubt the validity of the review’s consideration in quantifying and comparing the advantages and disadvantages. Can advantage and disadvantage from different respects be judged in quantity and compared with each other in such a simple way? Furthermore, even they could be calculated in quantity, it seems that the calculative way could not be that simple. For example, as to the judgment “chromosomes are four times longer than unit ribozymes, which leads to a four-fold disadvantage for chromosomes over unit ribozymes in terms of … decay” (in fact judgments like this “originated” very early [8]) – if the probability of the break of an phosphodiester bond (*P*_*BB*_) in a time step is 0.1, then after a time step, for an 8-nt unit ribozyme, the probability that it remains intact is (1–0.1)^(8–1)^, i.e. about 0.48; for an 32-nt chromosome, the corresponding probability is (1–0.1) ^(32–1)^, i.e. about 0.,038. Thus, the disadvantage of the chromosome seems to be already over 12-fold. While we enjoy ourselves on this calculative way which seems to be more clever and rigorous, suddenly we found that, in this way, the quantity about the disadvantage may change dramatically accompanying the value alteration of *P*_*BB*_. Which would be a more appropriate way? It seems difficult to envision one at once. The calculation of the advantage concerning the replication would be more sophisticated, if still feasible.

If the reviewer’s consideration of advantage-disadvantage quantification and comparison is problematic, his consequent consideration for the interpretation to the “phenomenon” that “chromosomes must have an extremely large additional advantage provided by the circular structure in order to survive in a protocell system” would loss its point of origin. However, indeed, according to our inference in the Background section and the result of the computer simulation, the advantage for a chromosome in respect of assortment load (i.e. avoid gene-loss during protocell division) [8] is not enough to counterbalance those apparent disadvantages for it. This is just the central concern of the present work. At least, in the beginning this is the case – in the long run, the merit of the chromosome concerning the reduction of the assortment load would certainly ascend accompanying the increase of the gene number, which would then strengthen the existence of the chromosomes as a result of evolution. Admittedly, in this study we cannot discern exactly how much the advantage concerning reducing the assortment load take its role, what we show here is this advantage should be insufficient and the strategy of circularity and self-cleavage may have been important.

By the way, there are two misunderstandings here. First, *P*_*BB*_ would act on each phosphodiester bond, whereas *P*_*NDE*_ would act only on terminal nucleotide residues. Therefore, we cannot say “chromosomes decay nearly one order of magnitude slower than unit ribozymes (*P*_*BB*_ < <*P*_*NDE*_)”. Second, self-cleaving effect would not occur in the circular form of the chromosome, as mentioned in text (the second paragraph of the Result section and the legend of Figure 1; see also our response to reviewer 1). Therefore, the reviewer’s judgment that “the primitive transcription based on self-cleavage incurs a large fitness cost to chromosomes, … because self-cleavage entails self-destruction” should not be the case in our model system. Perhaps, our title should, in a more rigorous form, be “A chromosome adopting a strategy of circularity in itself and self-cleavage in its linear transcripts may have emerged in the RNA-based protocell”; however, this would seem too long and complex for a title.

#### *Reviewer comments*

As regards the second question (i.e., the evolutionary origin of primitive chromosomes), the authors consider that primitive chromosomes occasionally appear through random ligation, recombination, and circularization of ribozymes by chance (Discussion section). This assumption might not be reasonable because there seems to be a large gap in terms of complexity between the assumed chromosomes and unit ribozymes. This assumption can be (and should be) tested by the current model, which seems to incorporate all the elementary steps that are necessary for the emergence of first primitive chromosomes in a chromosome-less system (viz., ligation, circularization, and sequence evolution). What would happen if simulations are started without assuming primitive chromosomes at the beginning?

#### *Authors’ response*

Indeed, the evolutionary origin of primitive chromosomes is a problem. However, it is another problem. Our topic here is “if a chromosome has come into being, could it sustain in the context of those mentioned difficulties? Our results showed that if the chromosome adopted a strategy of circularity and self-cleavage, it may overcome the difficulties, spreading in the system. Admittedly, to be a sufficient condition, the problem of the evolutionary origin of the chromosome should be demonstrated.

In the current model, we introduced four unit ribozymes for the sake of “a more representative form of a chromosome with linked genes” (mentioned in the first paragraph of the Results section). The evolutionary origin of such a chromosome from a system with the four unlinked ribozymes seems indeed a large gap. However, in reality, things may not have to occur this way, in one step. For example, in a primitive system with only two kinds of functional ribozymes favoring the protocell containing them (e.g. Rep and Nsr; see one of our previous reports [7] for such a system), a linear RNA with the two unit ribozymes might arise by random ligation/recombination, and then form a circular one by cyclization. This simplest circular RNA chromosome may survive in the system because it adopts the strategy of circularity and self-cleavage, as well as it benefits the protocell contain it in respect of assortment load during cell division. Then, by “gene duplication” (e.g. via an occasional ligation of two linear transcripts of the two-gene chromosome and the subsequent cyclization), a larger RNA molecule with four unit ribozymes (two couples of the original genes) may form. Then by some mutations, an RNA chromosome containing the four different unit ribozymes may appear and spread, for the new kinds of genes (i.e. Npsr and Asr [20]) can favor the protocells containing them.

Indeed, as the reviewer said, assumptions like this concerning the evolutionary origin of the chromosome may be tested by our model, which have incorporated all the elementary events that are necessary for the emergence of first primitive chromosomes in a chromosome-less system. At least, when there are only two kinds of unit ribozymes in the system, it may not be quite difficult. In fact, in our previous studies using a similar approach, such simulations about the appearance of the first functional RNA sequence (e.g. of Rep [18], Nsr [19] and Asr [20]) within the system were conducted. The successful de novo emergence (the appearance of the first sequence and its subsequent proliferate/spread) would largely rely on the number of the random seeds that we would like to test for the simulation and the time we would like to wait for the computation. When the object of our simulation and the model system become more complex, the study of the appearance of the first sequence would become very time consuming. Having recognized this, as well as the distinction between the problem of “the first appearance” and that of “the subsequent proliferate/spread”, in the later studies concerning more complicated problems, like in [7] and this work, we focused on the “proliferate/spread” problem, regarding “the first appearance” somewhat as “a game of chance and time”.

For the present case, if you insists to ask “What would happen if simulations are started without assuming primitive chromosomes at the beginning?” according to our experience, it is likely that nothing would happen – within your patience. There are two solutions: one is “to be more patient”; the other is to imagine the large gap as smaller intermediate gaps in a process of ongoing evolution (as mentioned above), and then test the smaller gaps with modified models.

If we explain these issues in the main text, apparently, it would seem too verbose. Therefore, thanks for the reviewer’s insightful comment on this point and certainly, also the chance offered by the journal for us to show our response to this comment.

#### *Reviewer comments*

I have a few more comments. First, the authors assume that circularization destabilizes the folding of RNA molecules and thereby renders them more suitable as templates. However, such destabilization might depend on the length of RNA molecules: a circular RNA might behave nearly as a linear molecule (at least locally) if its length is sufficiently long. Given that viroids are only a few hundred nucleotides long, which is about the same length as the template-directed RNA polymerase ribozyme [34], one needs to be cautious about generalizing the effect of circularization to chromosome-like molecules, which are expected to be longer than a few hundred nucleotides.

#### *Authors’ response*

As we mentioned above in our response to Reviewer 1, this opinion, as well as the one appeared in the last comment of Reviewer 1, implies that the circular RNA chromosome may have been short. As to the length of the polymerase ribozyme [34], we have to say that the effort is still an ongoing one. Perhaps in future we will construct a much shorter one that can act as a true replicase (able to replicate itself). Even if it turns out that any polymerase ribozyme has to be very long, the preliminary replicase ribozyme may have only been a ligase – short enough and loosely bound to the template-substrate complex – as demonstrated in our computer simulation work concerning the replicase [18] (actually just being the Rep in the current model).

Furthermore, we would like to talk a little more about the role of circularity to avoid the folding of RNA molecules. In fact, this role may have been somewhat stronger than the reviewer envisioned. For an efficient ribozyme, the correct folding should be very important, just like a protein enzyme. As mentioned in the text, those linked unit ribozymes in a chromosome might interfere with each other in folding, rendering the chromosome a better template. However, the two unit ribozymes at the two ends of a linear chromosome would still have a free “tail” each, which might fold “inwards” to the chromosome chain and entail the in-situ formation of the “correct” (compact) structures of these two unit ribozymes. Considering template-directed copying is perhaps more likely to start from the chain ends, this impact may have been significant. That is, circularity may also have been important in respect of avoiding this impact, by “removing the free tails”. This effect, beyond the pure steric impediment caused by the circularity, is apparently not influenced by the length of the RNA chromosome.

#### *Reviewer comments*

Second, the authors argue that terminal phosphodiester bonds are more sensitive to hydrolysis than internal (i.e., within-chain) phosphodiester bonds because terminal base stacking is less stable than internal base stacking. This argument seems to imply that circular molecules are more vulnerable to hydrolysis than linear molecules because, as the authors assume, circularization destabilizes the folding of RNA molecules. But, this is the opposite of the assumed effect of circularization.

#### *Authors’ response*

This point belongs to those issues difficult to clarify only by deduction. For example, it may be argued that though circular molecules, in a single chain form, might be more vulnerable to hydrolysis than linear ones, they are actually more likely to appear in a double chain form since they may act as better templates than linear ones. Apparently the base-stackings in a double chain would be more (and stronger) than those in a single chain – no matter how much it tends to self-fold.

#### *Reviewer comments*

Third, the model seems to assume that the existence of ribozymes changes the equilibrium constants of chemical reactions such as the conversion reaction between nucleotide precursors and nucleotides. Is this reasonable?

#### *Authors’ response*

No, we have not assumed that the existence of ribozymes changes the equilibrium constants of chemical reactions. For example, the rate that a nucleotide precursor forms a nucleotide is represented by *P*_*NF*_, whereas the rate that a nucleotide decays into a nucleotide precursor is represented by *P*_*ND*_. When a nucleotide synthetase ribozyme (Nsr) exists and acts upon a nucleotide precursor, the rate that this precursor would transform into a nucleotide is *P*_*NFR*_ (> > *P*_*NF*_). Indeed, it seems here that the equilibrium constant is changed. However, the reaction catalyzed by the Nsr is actually not the original non-enzymatic reaction. The reactive path has been changed by the incorporation of some high-energy factor, just like in modern organisms (e.g. ATP as a common high-energy factor). If enzymatic reactions are the same as the original non-enzymatic reactions, that is, degradations are speeded as well as syntheses, how could our “living” system be sustained? The reason why here equilibrium constants appear to have been changed by ribozymes is that the events concerning the potential high-energy factor were omitted in our model – “the energy problem was simplified in the simulation…”, as stated in the earliest paper of our group that reported the usage of this kind of Monte-Carlo model system to simulate early evolution in the RNA world [35].

### Reviewer 3: Dr. Eugene Koonin, National Center for Biotechnology Information, NIH, USA

#### *Reviewer comments*

Here Wentao Ma and colleagues continue their series of modeling studies of various aspects of the putative primordial RNA world. The current installment is based on the old idea of Diener that viroids are relics of the RNA world. Certainly, this is an attractive idea given that these are indeed the simplest known replicators, and they are stable small RNAs which is important for RNA World models. The new idea introduced by Wentao Ma and colleagues is the self-cleavage of a viroid-like circular RNA molecule into smaller molecules with various potential activities, via the action of built-in hammerhead ribozymes. There is no precedent for this among modern RNAs but neither is there anything biologically or chemically implausible about this model. There is actually little doubt that such a molecule can be produced experimentally, and its properties will be of interest. Certainly, a merit of the model.

#### *Authors’ response*

Thanks to the reviewer for the remark. In particular, indeed, such a molecule might be produced experimentally in future, and its properties will be interesting.

#### *Reviewer comments*

Wentao Ma then proceeded to develop a Monte Carlo-type model of a replicator system consisting of such viroid-like molecules and claim that the result revealed robustness and viability of this type of replicators systems. This reviewer's attitude to this kind of models is ambivalent. On the one hand, it is clear that (almost) everything depends on parameterization, and with some combinations of parameters, nearly any outcome is possible. On the other hand, the model is not useless because it shows that such replicator systems can exist in a large domain of the parameter space.

#### *Authors’ response*

Yes, the number of parameters in the model is great, and this may bring about an impression that any outcome of the simulation may be possible, depending on the values of the parameters. We would like to defend this situation from three aspects. First, as the reviewer mentioned, “the model is not useless because it shows that such replicator systems can exist in a large domain of the parameter space” (see the Additional file 1: Figures S2-S5), especially considering the domain is consistent with a serial of considerations concerning the relation of the parameters in logic and in real situations (see those descriptions in the Method section). Second, the introduction of so many parameters was due to the complexity of the system we intended to model. For example, in our early studies [18-20], in which the target issue was much simpler, the number of parameters was much smaller. Indeed, in other computer simulations (using different methods) in the field, parameters could be much fewer; however, none of them (as far as we know) could be used to tackle so complicated a system as the current one. Third, according to our experience, it is actually not the case that everything depends on parameterization. For example, if circularity or self-cleavage is not assumed as the feature of the RNA chromosome, you can hardly expect any “positive result” concerning the spread of the RNA chromosome in the model system, no matter how you manage to adjust (the other) parameters. In other words, a positive result concerning the target issue is often difficult to achieve unless you make some key assumptions. Another example may come from our previous study concerning the spread of an RNA replicase ribozyme (just the Rep in the present study; already mentioned in our response to Reviewer 2′s comments) [18], which had been supposed to be the first replicator in the RNA world. It was shown in that study that the Rep can hardly spread in a random RNA pool (in a naked circumstance, not within protocell), unless it is a short, loosely template-binding ligase (note: not a polymerase). Indeed, our studies of this series were just aimed at revealing those crucial assumptions and key parameters that might brought a “positive” outcome, which usually referred to the successful proliferation/spread of some target replicator(s) in the model system. In this context, a “positive” outcome should be “robust” in a large space domain of the other parameters, as already shown in each of these studies.

#### *Reviewer comments*

So on the whole, this is an interesting hypothesis. I am surprised Wentao Ma and colleagues do not mention Hepatitis Delta virus which is the largest known replicating circular RNA molecule and encodes a protein, thus perhaps resembling the primordial genomes that bridged the RNA World and the more advanced RNA-protein World.

#### *Authors’ response*

Here the reviewer raises an interesting point. In fact, the circular RNA chromosome of Hepatitis Delta virus (HDV) contains a viroid-like region and a protein-coding region [36,37]. Its replication is similar to that of the viroid, in which a self-cleavage ribozyme (residing within the viroid-like region) plays a key role. Is the HDV a relic of the RNA-protein World, which implies that the strategy of circularity and self-cleavage could have “sustained” to such an advanced world? This may have been the case (what an exciting case!), if we would like to put aside the doubts about the length of the circular RNA chromosome (i.e. it may have been short; see associated comments from the other two reviewers and our response) – a chromosome comprising all the genes for a translation machine may have been quite long. Considering that a long circular RNA chromosome is “still possible” because circularity may have prevent an RNA chromosome’s self-folding by “getting rid of” free ends rather than by pure steric impediment (see our response to the second reviewer on this point), the exciting case may really have been the case. Who can say definitely not?

#### *Reviewer comments*

Thus, there is nothing overtly wrong with the scenario put forward by Wentao Ma and colleagues, and moreover, I think it is more ingenious than many other models that circulate in the origin of life field. Except perhaps for one fundamental issue: all of this conceivably could have happened as outlined in the model, only there is not a shred of evidence it actually happened this way. A general problem with models of primordial evolution, not to be held against the authors.

#### *Authors’ response*

Thanks to the reviewer for his praise. As to the fundamental issue mentioned, we agree with the reviewer. But we have an additional remark about this issue. The origin of life is a problem of history. The process occurred in such remote past that perhaps we will never know exactly what happened actually. But perhaps this does not matter. Our scientific studies on this history are largely (if not completely) aimed at associated rules. In fact, rules behind phenomena are the shared concern of all scientific fields. Compared with how an event in this process may have happened, we are, in some degree, more interested in how it could have happened. Certainly, if we could know quite a few plausible ways for the event, we would be more interested in whether it happened this way or that way in reality. Unfortunately, this is usually not the case in the field of the origin of life. Therefore, in general, on the one hand we should try to collect evidence relevant to the process, but on the other we should appreciate that any plausible mechanisms we find concerning possible events in this process would be quite valuable.

## Competing interests

The authors declare that they have no competing interests.

## Authors’ contributions

WTM conceived of the study, did the simulation work and wrote the manuscript. CWY and WTZ participated in the simulation work. All authors read and approved the final manuscript.

## Supplementary Material

Additional file 1: Figure S1-S5 Five supporting figures for the paper. Click here for file
