# Supplementary material for: Circularity and self-cleavage as a strategy for the emergence of a chromosome in the RNA-based protocell
Source: Biol Direct. 2013 Aug 23;8:21. doi: 10.1186/1745-6150-8-21 (PMC3765326; doi:10.1186/1745-6150-8-21)
Supplement: Additional file 1: Figure S1-S5 — Five supporting figures for the paper. [file 1745-6150-8-21-S1.pdf]

Five supporting figures (Fig. S1-S5) for

Circularity and self-cleavage as a strategy for the emergence of a chromosome in the RNA-based protocell

by Wentao Ma*, Chunwu Yu, and Wentao Zhang

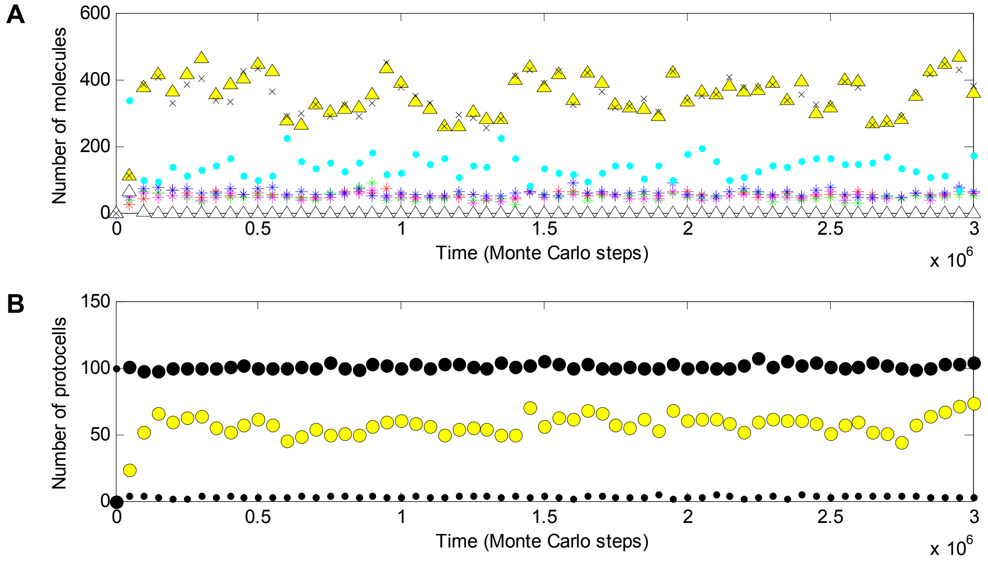

Figure S1. Representative case of the spread of the chromosome when the antisense chain is inoculated. (***A***) At the molecular level. (***B***) At the cellular level. The interpretation of this figure is the same as for Fig. 2, except that the chromosome molecules inoculated (at step 1×10^4) are the antisense chains instead of the sense chains. The parameter values are identical to those for the case shown in Fig. 2.

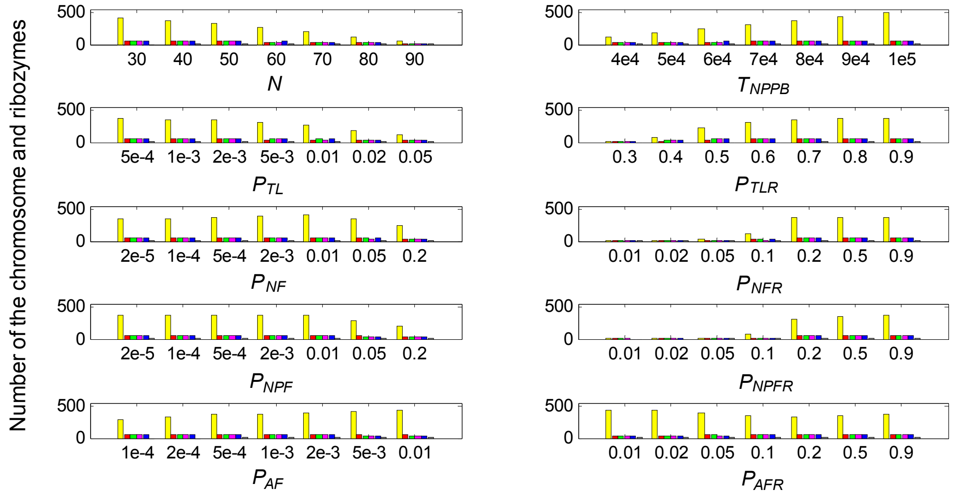

Figure S2. Influence of the parameters on the spread of the chromosome (part 1). For the 43 parameters listed in Table 1, the influence of three of them (P_{CRTT}, P_{LRTT}, and F_{IB}) is shown in Fig. 4B, and the influence of the other 40 is shown in Figs. S2-S5. See the legend to Fig. 4B for the interpretation of the colored bars.

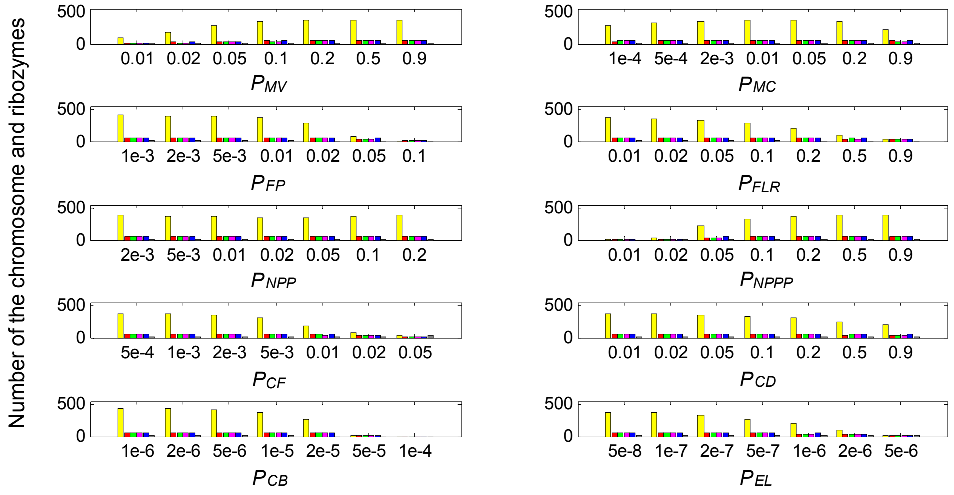

Figure S3. Influence of the parameters on the spread of the chromosome (part 2).

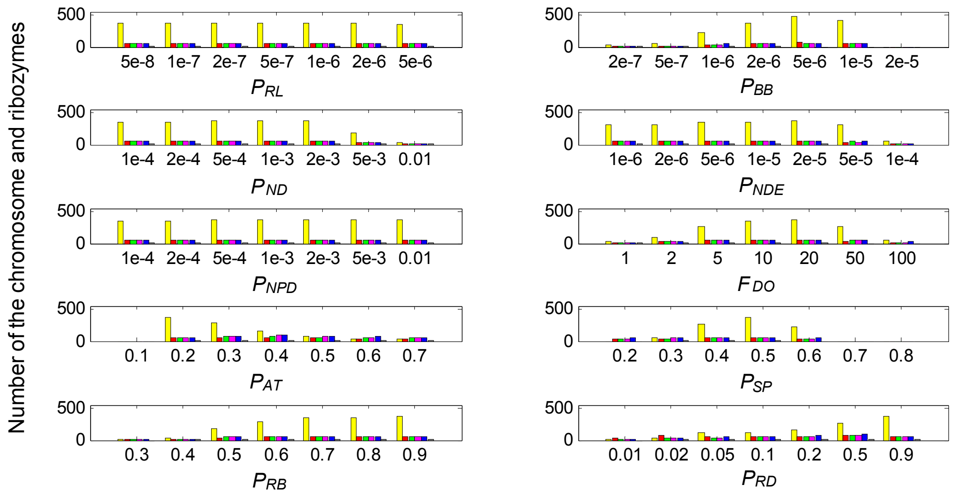

Figure S4. Influence of the parameters on the spread of the chromosome (part 3).

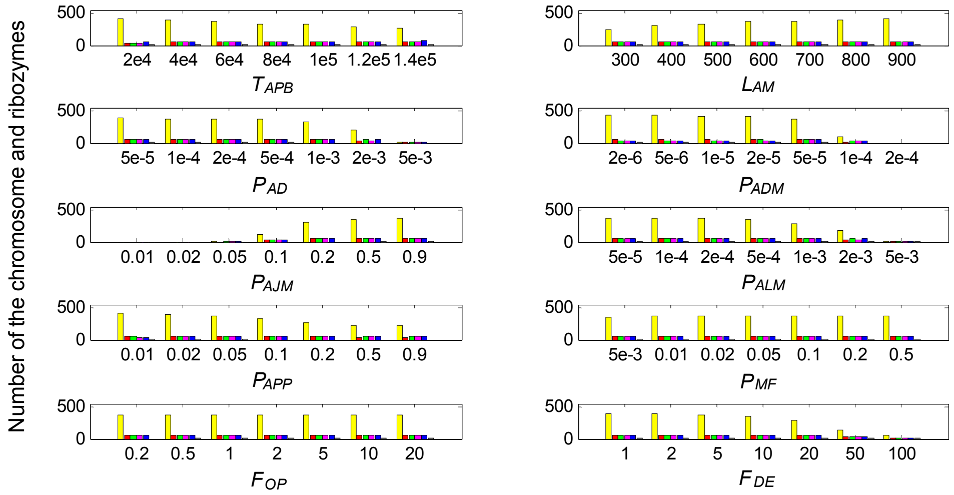

Figure S5. Influence of the parameters on the spread of the chromosome (part 4).
